# Bayesian network meta-analysis of face masks' impact on human physiology

**DOI:** 10.1038/s41598-022-09747-z

**Published:** 2022-04-06

**Authors:** Kamil Litwinowicz, Marcin Choroszy, Maciej Ornat, Anna Wróbel, Ewa Waszczuk

**Affiliations:** 1 Department of Biochemistry and Immunochemistry, Wroclaw Medical University, Wrocław, Poland; 2 Department of Microbiology, Wroclaw Medical University, Wrocław, Poland; 3 Department of Human Morphology and Embryology, Wroclaw Medical University, Wrocław, Poland; 4 Department of Psychotherapy and Psychosomatic Diseases, Wroclaw Medical University, Wrocław, Poland; 5 Department of Gastroenterology and Hepatology, Wroclaw Medical University, Wrocław, Poland

**Keywords:** Medical research, Disease prevention, Occupational health, Public health

## Abstract

Several concerns regarding the safety of face masks use have been propounded in public opinion. The objective of this review is to examine if these concerns find support in the literature by providing a comprehensive overview of physiological responses to the use of face masks. We have performed a systematic review, pairwise and network meta-analyses to investigate physiological responses to the use of face masks. The study has been registered with PROSPERO (C RD42020224791). Obtained results were screened using our exclusion and inclusion criteria. Meta-analyses were performed using the GeMTC and meta R packages. We have identified 26 studies meeting our inclusion and exclusion criteria, encompassing 751 participants. The use of face masks was not associated with significant changes in pulsoxymetrically measured oxygen saturation, even during maximal-effort exercises. The only significant physiological responses to the use of face masks during low-intensity activities were a slight increase in heart rate, mildly elevated partial pressure of carbon dioxide (not meeting criteria for hypercarbia), increased temperature of facial skin covered by the mask, and subsequent increase of the score in the rating of heat perception, with N95 filtering facepiece respirators having a greater effect than surgical masks. In high-intensity conditions, the use of face masks was associated with decreased oxygen uptake, ventilation, and RR. Face masks are safe to use and do not cause significant alterations in human physiology. The increase in heart rate stems most likely from increased respiratory work required to overcome breathing resistance. The increase in carbon dioxide is too small to be clinically relevant. An increased rating of heat perception when using face masks results from higher temperature of facial skin covered by the mask.

## Introduction

As of March 2021, COVID-19 has caused more than two million deaths worldwide^1^. With the limited availability of vaccines, strategies that reduce the rate of infection continue to play a pivotal role in minimizing the mortality and strain of COVID-19 on the healthcare sector. Among these strategies, public use of face masks has been a topic of heated debate. Wearing a face mask is effective in reducing the incidence of several betacoronavirus infections, including SARS-CoV-2^2^. The World Health Organization advises public use of face masks^3^. In line with these guidelines, various countries introduced laws enforcing mask use by the general population^4^. However, the unprecedented surge in the use of face masks has been associated with the emergence of several concerns regarding their safety and effects on physiology, especially during high-intensity exercises^5^.

Several hypotheses of how face masks could exert harmful effects have been brought up. The most commonly mentioned, both in scientific writing and public opinion, are reduction of available oxygen and increased dead space with subsequent hypercapnic hypoxia^5^. These hypotheses are not completely unfounded in literature, for example, Beder et al.^6^ reported that oxygen saturation measured with a pulse oximeter (SpO_2_) of surgeons operating in surgical masks was decreased. However, the lack of a proper control group precludes making firm conclusions. In addition, Lim et al.^7^ and Geiss^8^ point to CO_2_ retention as a likely cause of headaches commonly associated with wearing personal protective equipment (PPE). The literature on the topic reports some conflicting results—for example, Kim et al.^9^ report a significant increase in respiratory rate (RR) when wearing an N95 filtering facepiece respirator (FFR), while Fikenzer et al.^10^ report a significant decrease in RR. The wealth of original research on the topic, with different designs (e.g. taking measures during low or high-intensity activities), examining different types of face masks, and aforementioned reports of conflicting results warrants systematic synthesis, with an exploration of causes underlying discrepancies. Although a meta-analysis on this topic has been recently published^11^, it did not evaluate the differences in physiological responses to the mask use depending on the intensity of the testing protocol and did not perform network meta-analysis which would allow comparing the effects of N95 and surgical masks^12,13^. The goal of this manuscript is to fill this gap and to provide a comprehensive evaluation of the face masks and disposable FFRs effects on human physiology during low, moderate, and high-intensity activities.

## Results

**Figure 1:**
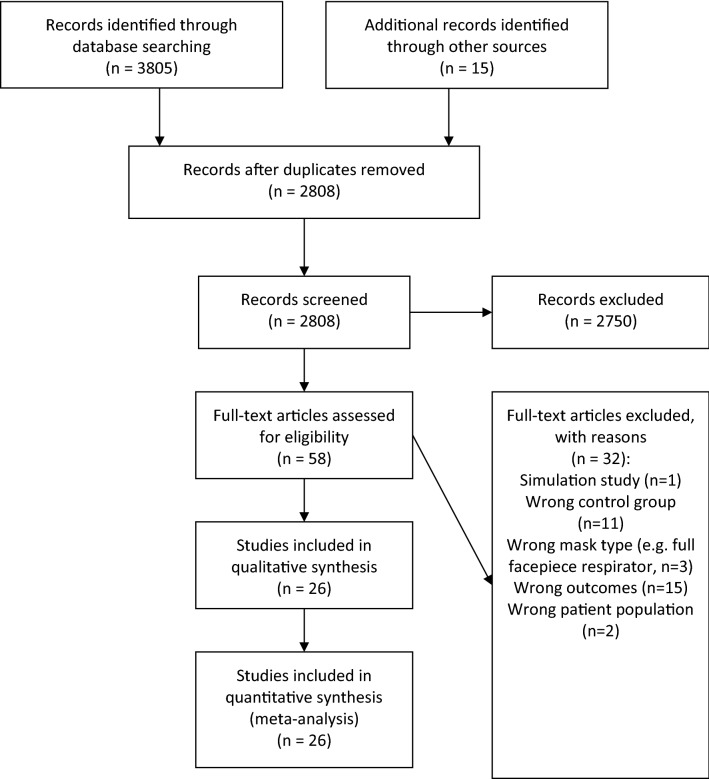
Flow diagram of the study selection process.

**Figure 2:**
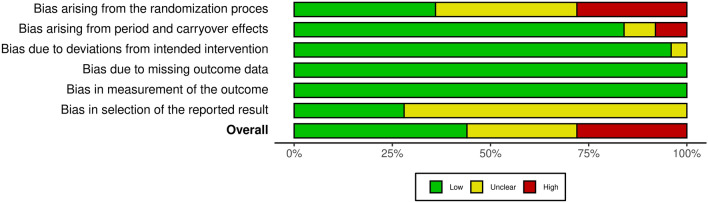
Risk of bias assessment for cross-over trials (based on the second version of Cochrane risk of bias tool).

We have identified 26 studies (25 cross-over and one retrospective observational study), encompassing a total of 751 participants (Fig. 1, Table 1). Overall, 11 crossover studies were rated as having a low risk of bias, 7 as moderate, and 7 as high. Bias arose most commonly from a lack of proper randomization (Fig. 2 and Supplementary Fig. 1). Newcastle–Ottawa Scale assessment of non-randomized studies is provided in Supplementary Table 1. The geometry of comparisons is depicted in Supplementary Fig. 2, with a line thickness corresponding to the sum of participants in comparison, and a dotted line denoting indirect comparison. The majority of comparisons were fully connected (i.e. direct comparisons of all conditions were available).

**Table 1:** Characteristics of the included studies; *HR* heart rate, *RR* respiratory rate, *SpO*_*2*_ pulsoxymetrically measured oxygen saturation, *tPCO*_*2*_ transcutaneous CO_2_ measurement, *RPE* perception of exertion, *RHP* rating of heat perception, *SOIIBT* superomedial orbital infrared indirect brain temperature, *SBP* systolic blood pressure, *DBP* diastolic blood pressure, *IET* incremental exertion test, *N* sample size.

Study	Design	N	N95	Surgical	Reported outcomes
Lässing 2020^14^	Cross-over trial	14	−	+	HR, RR, SBP, DBP, SV, RPE, SpO_2_, spirometry
Carrizal and Rodriguez 2020^15^	Retrospective cohort study	180	−	+	HR, SBP
Chen et al. 2015^16^	Cross-over trial	15	+	−	RR
DiLeo et al. 2017^17^	Cross-over trial	18	+	−	RR, facial and aural temperatures, SOIIBT, RHP
Epstein et al. 2020^18^	Cross-over trial	16	+	+	HR, RR, SBP, SpO_2_, etCO_2_, IET
Fikenzer et al. 2020^10^	Cross-over trial	12	+	+	HR, SBP, DBP, SV, RR, spirometry, PCO_2_, pH, IET, RHP
Jones 1991^19^	Cross-over trial	67	+	−	HR, RR, SBP, DBP
Kim et al. 2013^9^	Cross-over trial	20	+	−	HR, RR, SpO_2_, tPCO_2_
Kim et al. 2014^20^	Cross-over trial	67	+	−	Aural temperature
Laird et al. 2002^21^	Cross-over trial	18	+	−	HR, facial temperature
Li et al. 2005^22^	Cross-over trial	10	+	+	HR, facial temperature, RHP
Li et al. 2021^23^	Cross-over trial	10	−	+	Spirometry, HR, RPE
Luximon et al. 2016^24^	Cross-over trial	20	+	+	Facial temperature, RHP
Mapelli et al. 2021^25^	Cross-over trial	12	+	+	HR, RR, SpO_2_, SBP, DBP, tPCO_2_, RPE, spirometry
Ramos-Campo et al. 2020^26^	Cross-over trial	14	−	+	HR, RPE
Roberge et al. 2010^27^	Cross-over trial	10	+	−	HR, RR, SpO_2_, TV, tPCO_2_
Roberge et al. 2012a ^28^	Cross-over trial	20	−	+	HR, RR, SpO_2_, tPCO_2_, Core and facial temperatures, RPE, RHP
Roberge et al. 2012b ^29^	Cross-over trial	10	+	−	Core and facial temperatures
Roberge et al. 2014^30^	Cross-over trial	22	+	−	HR, RR, SpO_2_, tPCO_2_, aural temperature, RPE, RHP
Scarano et al. 2020^31^	Cross-over trial	20	+	+	Facial temperature, RHP
Serin et al. 2020^32^	Cross-over trial	48	+	+	HR, RPE
Shaw et al. 2020^33^	Cross-over trial	23	−	+	HR, SpO_2_, IET
Shein et al. 2021^34^	Cross-over trial	50	−	+	HR, SpO_2_, tPCO_2_
Spang and Pieper 2020^35^	Cross-over trial	12	+	−	SpO_2_
Wong et al. 2020^36^	Cross-over trial	23	−	+	HR, RPE
Yip et al. 2005^37^	Cross-over trial	20	+	+	Aural temperature

**Figure 3:**
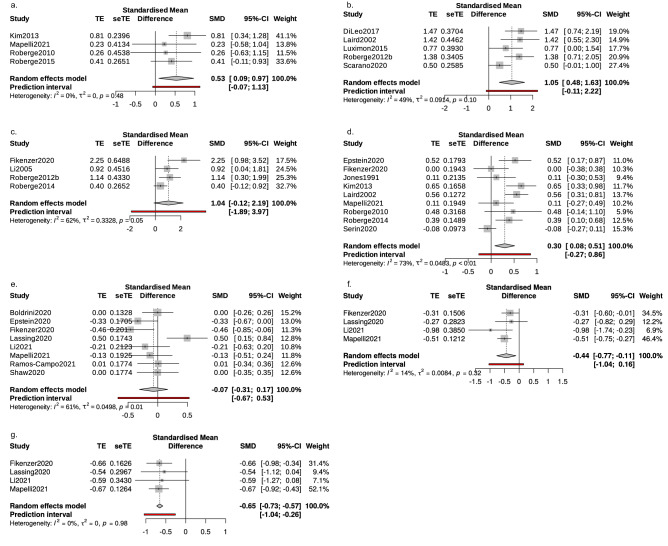
Statistically significant pairwise comparisons; Effect of N95 filtering facepiece respirators on (**a**) transcutaneous carbon dioxide pressure, (**b**) temperature of facial skin covered by the mask, (**c**) subjective rating of heat perception, (**d**) heart rate. Effect of surgical mask on: (**e**) respiratory rate, (**f**) VO_2max_/kg and (**g**) VE during high intensity activity.

**Figure 4:**
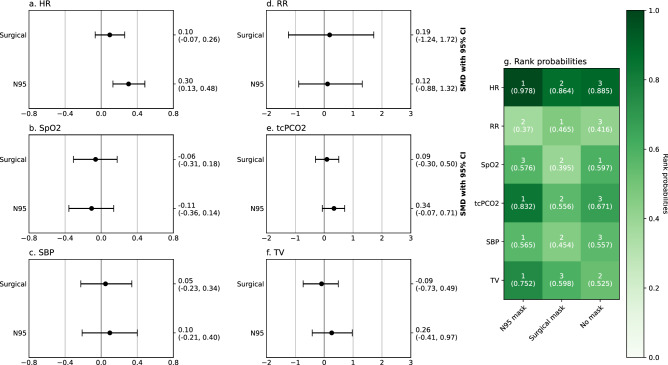
Network meta-analysis of N95 filtering facepiece respirators and surgical masks’ effect on physiological outcomes during low-intensity activities. Numbers in rank probabilities heatmap represent rank with the highest probability and corresponding probability for given measure and condition; *HR* heart rate, *SpO*_*2*_ pulsoxymetrically measured oxygen saturation, *SBP* systolic blood pressure, *RR* respiratory rate, *tcPCO*_*2*_ transcutaneous carbon dioxide pressure, *TV* tidal volume.

**Figure 5:**
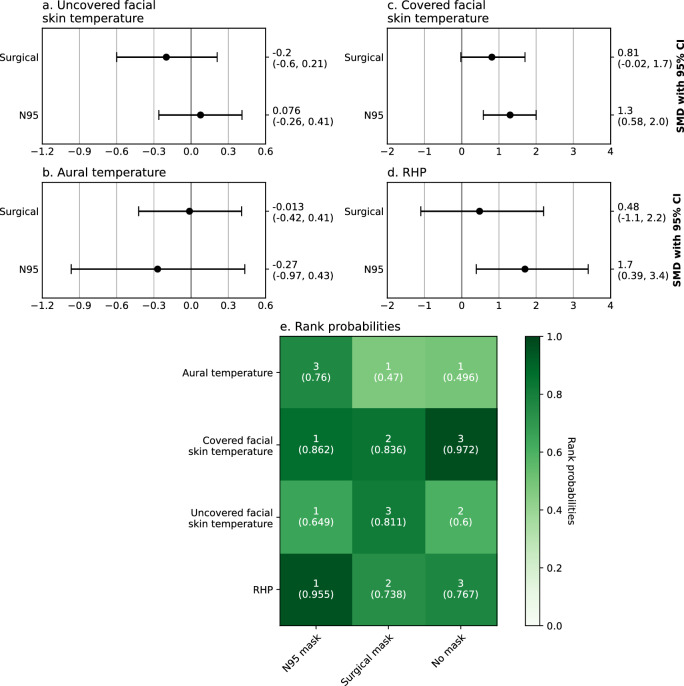
Network meta-analysis of N95 filtering facepiece respirators and surgical masks’ effects on thermoregulation. Numbers in rank probabilities heatmap represent rank with the highest probability and corresponding probability for given measure and condition; *RHP* a subjective rating of heat perception.

**Figure 6:**
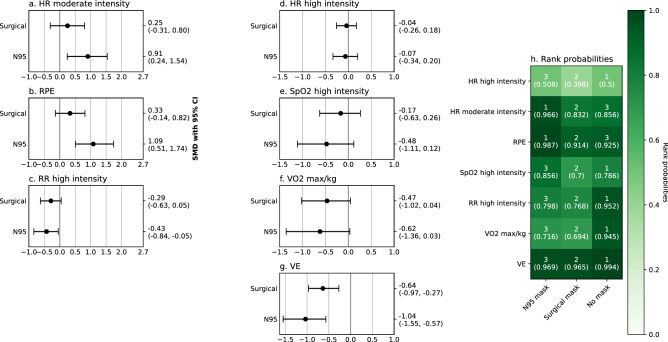
Network meta-analysis of N95 filtering facepiece respirators and surgical masks effects on physiological outcomes during moderate and high-intensity exercises. Numbers in rank probabilities heatmap represent rank with the highest probability and corresponding probability for given measure and condition; *RPE* a perception of exertion, *HR* heart rate, *VE* ventilation, *VO*_*2*_ oxygen uptake.

We have performed eighteen pairwise meta-analyses (Supplementary Fig. 3) examining the effect of surgical masks and N95 FFRs on heart rate (HR), respiratory rate, pulsoxymetrically measured oxygen saturation (SpO_2_), tidal volume (TV), transcutaneous carbon dioxide pressure (tcPCO_2_), systolic blood pressure (SBP), and various measures related to thermoregulation (aural temperature, facial skin temperature covered or not covered by a mask, and subjective rating of heat perception (RHP)) during low-intensity activities. Statistically significant comparisons are depicted in Fig. 3. Seven additional pairwise meta-analyses (Supplementary Fig. 3) were performed examining the effects of N95 FFRs and surgical masks on HR, RR, TV, oxygen uptake (VO_2_), ventilation (VE), and on the perception of exertion (RPE) during moderate or high-intensity exercise. To compare the sizes of the effect of surgical masks and N95 FFRs seventeen network meta-analyses were performed (Figs. 4, 5, 6). Outcomes that were reported by at most two studies meeting our inclusion criteria are described below as a narrative part of our systematic review.

### Low-intensity activities

The only significant results of pairwise comparisons regarding cardiovascular and pulmonary outcomes were a small increase in HR (standardized mean difference (SMD) 0.30, confidence interval (CI) 0.08; 0.51) and tcPCO_2_ (SMD 0.53, CI 0.09; 0.97) when wearing an N95 FFR (Fig. 3). Consistently, one of the papers obtained in the systematic review (Epstein et al.^18^) reported that wearing N95 FFR was associated with an increase in end-tidal carbon dioxide, both at rest and during high-intensity exercise (44 mmHg for N95 vs 35 mmHg without a mask during high-intensity exercise and 39 mmHg vs 41 mmHg during rest). A similar but less pronounced effect was found for surgical masks (40 vs 35 mmHg). However, in our pairwise comparisons, surgical masks did not significantly affect any outcome during low-intensity activities (Supplementary Fig. 3). Using N95 FFRs did not significantly influence tidal volume during low-intensity activities (Supplementary Fig. 3s).

Wearing an N95 FFR significantly increased RHP (SMD 1.04, CI − 0.12; 2.19, Fig. 3c) and facial skin temperature in areas covered by the mask (SMD 1.05, CI 0.48; 1.63, Fig. 3b). No significant differences in temperature of facial skin not covered by face masks or aural temperature were observed (Supplementary Fig. 3a,p). Two of the obtained studies^28,29^ reported that using face masks and FFRs does not significantly affect direct core temperature measures. One of the studies included in the systematic review^17^ examined the effect of N95 FFRs on superomedial orbital infrared indirect brain temperature (SOIIBT) and reported that the use of N95 FFRs is not associated with an increase in SOIIBT.

One of the most robust results of network meta-analyses was obtained for HR, where N95 FFR had a significantly greater effect (SMD 0.30, CI 0.13; 0.48) than surgical mask (SMD 0.1; CI − 0.07; 0.26) with rank probabilities over 0.9 for no mask and N95 FFR conditions and 0.864 for the surgical mask (Fig. 4a,g). In other comparisons only covered facial skin temperature and RHP produced high-rank probabilities: 0.972 for no-mask condition having the least effect on facial skin temperature and 0.955 for N95 FFRs having the greatest effect on RHP (SMD 0.79, CI 0.02; 2.0, Fig. 5). The forest plot showed highly overlapping confidence intervals for N95 FFRs and surgical masks.

### Moderate and high-intensity activities

Six studies^18,19,26,32,34,36^ examined effects of surgical masks or N95 FFRs on various physiological outcomes during moderate and nine^10,14,18,19,23,25,26,33,38^ during high intensity activities. Seven network meta-analyses examining the effects of face masks and FFRs on HR, RR, VO_2max_, VE, SpO_2,_ and RPE during high or moderate-intensity exercises were performed (Fig. 6). Five pairwise meta-analyses examining the effect of surgical masks on HR (Supplementary Fig. 3d), SpO_2_ (Supplementary Fig. 3r), VO_2max_/kg (Supplementary Fig. 3f), RR (Fig. 3e), and VE (Fig. 3g) during high-intensity activities were performed. One pairwise meta-analysis examined the effect of surgical masks on HR during moderate-intensity activities (Supplementary Fig. 3f). Two pairwise analyses were performed for the effect of N95 FFRs on HR during moderate and high-intensity activities (Supplementary Fig. 3c,e).

In network meta-analyses, high-rank probabilities were obtained for N95 FFRs having the highest effect on HR during moderate-intensity exercise (SMD 0.91; CI 0.11, 1.7; rank probability 0.928, Fig. 6a,h) and on RPE (rank probability 0.941, Fig. 6b,h). In addition, no mask condition was associated with the highest RR (rank probability 0.952, Fig. 6h) and VO_2max_ (rank probability 0.945, Fig. 6h). High rank probabilities were obtained for both N95 FFRs (SMD − 1.04; CI − 1.55, − 0.57) and surgical masks (SMD − 0.64; CI − 0.97, − 0.27) having a negative effect on VE and for N95 FFRs having a stronger effect than surgical masks (Fig. 6g,h).

Significant pairwise meta-analyses showed a small decrease of RR (SMD − 0.07; CI − 0.31, 0.17, Fig. 3e) during high-intensity activity, reduced VO_2max_/kg (SMD − 0.44; CI − 0.77, − 0.11, Fig. 3f), and reduced VE (SMD − 0.65; CI − 0.73, − 0.57) when wearing a surgical mask. Only two of the obtained studies^10,25^ reported effects of N95 FFRs on VO_2max_/kg and VE—both showed a significant negative effect. The results regarding differences in RR between no mask and N95 FFRs were conflicting—Epstein et al.^18^ reported no significant difference and Fikenzer et al.^10^ reported a significant reduction of RR associated with wearing N95 FFRs. Two of the studies included in the systematic review examined the effect of N95 FFRs on SpO_2_ during high-intensity exercises^18,25^. Neither reported significant differences between the mask and no-mask conditions. Further two studies meeting inclusion criteria examined the effect of face masks and FFRs on stroke volume (SV) and cardiac output (CO)—Lässing et al.^14^ reported no significant impact of the surgical mask on SV and CO; the same result was obtained for both surgical masks and N95 FFRs by Fikenzer et al.^10^. In addition, Fikenzer et al.^10^ reported that the use of N95 FFRs and the surgical mask does not affect blood gases. Time to exhaustion in incremental exertion test (IET) was assessed by two of the studies included in our systematic review. Fikenzer et al.^10^ reported a statistically significant reduction of time to exhaustion by 52 s caused by wearing N95 FFRs. The conflicting result was obtained by Epstein et al.^18^ who have reported no impact of N95 FFRs on IET results. Peak exercise workload was assessed only by one of the included studies (Mapelli et al.^25^); they have reported a negative effect of N95 FFRs on peak exercise workload. Mapelli et al.^25^ and Fikenzer et al.^10^ reported a significant reduction in tidal volume when wearing N95 FFRs (both studies) and a surgical mask (only Mapelli et al.^25^).

We have obtained only two studies examining cloth masks. Studies by Shaw et al.^33^ and Shein et al.^34^ reports that the use of cloth masks is not associated with significant changes in pulsoxymetrically measured blood oxygen saturation and heart rate during low, moderate, and high-intensity activities, and that it does not affect the performance in incremental exertion test.

### Heterogeneity, consistency, and publication bias

Egger’s test has shown significant funnel plot asymmetry in comparison of N95 FFRs and surgical masks’ effects on RHP (Supplementary Figs. 4 and 5). Node-splitting analyses did not show significant inconsistency for any of the network comparisons.

Several comparisons manifested significant heterogeneity (Supplementary Fig. 6). GOSH plots clustering and visual inspection of Baujat plots revealed, that two studies (Fikenzer et al.^10^ and Serin et al.^32^) were disproportionately responsible for this heterogeneity in most comparisons (Supplementary Figs. 7–14). Appropriate sensitivity analyses were performed.

### Sensitivity analyses

Rank probabilities, SMDs, and confidence intervals were not significantly altered by the imputation of various correlation coefficients (Supplementary Figs. 15–31). Additional sensitivity analyses were performed for the effects of face masks and FFRs on RR, where we have found an outlier study (Jones^19^). The exclusion of this study produced significantly narrower confidence intervals; however, rank probabilities were still low (Supplementary Fig. 32).

The exclusion of studies overly contributing to the heterogeneity did not significantly affect SMDs and CIs, with exception of N95 FFRs’ effect on RR (Supplementary Fig. 6). We hypothesize that the high influence of paper by Fikenzer et al.^10^ on heterogeneity in the analysis of N95 FFRs’ effect on RHP results from higher intensity level during the trial compared with other studies. Calculation of means and standard deviations from medians and quartiles provided by Serin et al.^32^ is the most likely explanation for high heterogeneity associated with this study (calculations were performed using the approach proposed by Wan et al.^39^).

## Discussion

The findings of our study show that wearing surgical masks or N95 FFRs slightly increases HR, tcPCO_2_, and covered facial temperature, with N95 FFRs having a greater effect than surgical masks. In addition, the use of surgical masks and N95 FFRs significantly reduced VO_2max_/kg and VE during high-intensity activity. No differences in other physiological variables were observed, most notably surgical masks and N95 FFRs did not significantly affect pulsoxymetrically measured oxygen saturation, even during submaximal and maximal intensity exercises.

Conflicting results regarding surgical masks and N95 FFRs’ effects on performance and RR during high-intensity exercise were obtained. This discrepancy may stem from different instrumentation used in studies—Fikenzer et al.^10^ and Mapelli et al.^25^ have used spirometry masks over N95 FFRs, which may have resulted in a much tighter fit of N95 FFRs than in normal use, thereby putting the external validity of this result into question. Reduction of VO_2max_ and VE caused by wearing N95 FFRs and surgical masks during submaximal effort was one of the most consistent results obtained in our study. Unfortunately, the measure of these outcomes requires the use of spirometry masks, which—as discussed above—might influence the results.

Slightly (but statistically significantly) higher HR associated with wearing N95 FFRs during low or moderate-intensity activities may be explained by higher respiratory work required to overcome increased breathing resistance^40^.

Several hypotheses point to CO_2_ retention as the main cause for the higher incidence of headaches when wearing N95 FFRs^7^. While our results show, that wearing N95 FFRs is associated with a slight increase in the partial pressure of carbon dioxide, the effect size was small and insufficient to cause hypercarbia (i.e. partial pressure of carbon dioxide exceeding 45 mmHg). This is in line with the results by Ong et al.^41^, who have shown that most of the headaches associated with the use of masks or FFRs meet diagnostic criteria of external compression headaches and that their location corresponds to points of pressure applied by the personal protective equipment (PPE). Hence, we conclude that not CO_2_ retention, but pressure caused by PPE is responsible for headaches associated with the use of FFRs.

Increased heat perception is frequently mentioned as the main cause for non-compliance in using PPE^42^. Several hypotheses attempted to explain why masks and FFRs are associated with higher heat perception, namely: increase in core temperature, brain warming, and increased facial skin temperature. The hypothesis regarding core warming is based on the fact that the respiratory tract is responsible for approximately 10% of total body heat loss^42^. However, one of our results is that the use of surgical masks or N95 FFRs is not associated with a significant increase in core temperature, both when approximated by aural temperature or when measured directly. The brain warming hypothesis was based on results from Cabanac et al.^43^ who have proposed that brain temperature significantly affects thermal comfort in humans. However, DiLeo et al.^17^ have found that using N95 FFRs does not significantly affect brain temperature. The final hypothesis points to the high density of thermoreceptors on facial skin^29^. Our results provide strong but indirect evidence for this hypothesis. We have found that using N95 FFRs is associated with increased temperature of facial skin covered by FFR, with a relatively high effect size of 1.05. None of the other objective outcomes associated with thermoregulation was significantly affected by the use of FFRs or surgical masks.

The strengths of this review include its comprehensiveness and the use of network comparisons. It is the first analysis providing a comprehensive, qualitative description of face masks and FFRs effects on physiology. The use of search terms maximized for sensitivity and subsequent scanning of the obtained papers' references has allowed us to identify a high number of studies relevant to the topic. The incorporation of network meta-analysis into our systematic review has allowed us to include several studies that would not be fit for classic, pairwise meta-analysis (i.e. studies comparing N95 FFRs to surgical masks).

Our study has several limitations. First of all, the longest trial of the included studies lasted only two hours. One might hypothesize that longer trials might result in a more pronounced effect of masks and FFRs. However, a study by Rebmann et al.^44^, which has evaluated the effects of N95 FFRs over 12-h nurses’ shifts is in agreement with our results (i.e. no effect on pulsoxymetrically measured oxygen saturation and a slight increase in the partial pressure of carbon dioxide, insufficient to cause hypercarbia). Another limitation is the study population. We did not consider studies on the children, however, a study by Goh et al.^45^ suggests that—similarly to the adult population—the use of N95 FFRs causes at the most very mild effect on the end-tidal carbon dioxide and does not significantly affect SpO_2_. In addition, we have excluded studies on participants with serious respiratory disorders. While the inclusion of this group might have resulted in increased applicability of our results to the broader population, we can not exclude that it would violate the transitivity assumption in network meta-analysis and paradoxically reduce the external validity of our study. Since one study reported that the use of N95 masks in patients with the chronic obstructive pulmonary disease was associated with changes in pulsoxymetrically measured oxygen saturation^46^, we would advise against extrapolating our results to this population. This highlights that research of face masks and FFRs’ effects on patients with respiratory disorders is urgently needed.

## Conclusions

The use of surgical face masks and N95 FFRs does not cause a reduction in pulsoxymetrically measured oxygen saturation, even during high-intensity exercise. N95 FFRs cause a slight accumulation of CO_2_, but this effect is not sufficient to cause hypercarbia. FFRs slightly increase HR, most likely due to increased respiratory work. Wearing N95 FFRs causes significant thermal discomfort, which stems most likely from an increased temperature of skin covered by FFR.

## Methods

The study adhered to guidelines outlined in the Preferred Reporting Items for Systematic Reviews and Network Meta-Analyses (PRISMA-NMA) statement^47^. The review protocol was registered at PROSPERO (CRD42020224791).

### Search strategy

We have performed a comprehensive literature search using keywords related to different types of face masks or FFRs (N95 respirators OR masks OR respiratory protective devices) and their effects on physiology (tidal volume OR respiratory rate OR partial pressure OR physical exertion OR heart rate OR body temperature OR skin temperature OR carbon dioxide OR blood pressure OR arterial pressure). The complete search strategy is provided in the Search strategy section of the Supplementary File. The search was performed on the following databases, from their inception to the final search date (20.12.2020): MEDLINE, EMBASE, CINAHL, Cochrane Central Register of Controlled Trials (CENTRAL), and WHO COVID-19 database.

### Inclusion and exclusion criteria

We have included studies examining physiological responses to the use of surgical masks, cloth masks, N95, N97, N99, FFP1, FFP2, or FFP3 FFRs in adults. Exclusion criteria included: respiratory failure, current use of oxygen therapy, heart failure, and pregnancy. Studies with multiple subgroups, where some met our inclusion criteria and others did not (e.g. pregnant and non-pregnant women) were considered only if they provided separate analyses of our subgroup of interest. The comparator group was either cohort not wearing any face mask or a cohort wearing a different type of face mask (e.g. surgical mask vs N95 FFR). Only studies published in English were considered. No publication date or publication status restrictions were applied.

### Assessment of eligibility and data extraction

Four authors performed a systematic review (KL, MC, MO, AW). After the removal of duplicates, obtained results were split into two parts. Abstracts and titles of manuscripts in each part were independently screened by two randomly assigned reviewers. Obtained studies were then read in full text and eligibility based on inclusion and exclusion criteria was determined. Discrepancies were resolved in the discussion panel of all manuscript authors. Data regarding study characteristics (title, author, study design, publication year, conflict of interest), participants, and outcomes were extracted.

### Quality assessment

The risk of bias of randomized controlled trials was assessed using the Cochrane risk of bias tool (version 2)^48^. For other study designs, the Newcastle–Ottawa Scale^49^ was used. Risk of bias plots were generated using the robvis tool^50^. To assess the publication bias, Egger’s test of funnel plot asymmetry was performed.

### Handling of cross-over and multi-arm trials

For cross-over trials, standardized mean differences (Hedge’s g) and corresponding standard errors were calculated from paired t-tests. If paired analyses were not reported in the given study, correlation coefficients borrowed from other manuscripts were imputed^51^. Correlation in multi-arm studies was accounted for by the network meta-analysis model employed in GeMTC^52^.

### Synthesis of results

Pairwise meta-analyses comparing given mask type against cohort not wearing any mask were performed for each outcome where at least three clinically homogenous studies were obtained. The comparisons were performed using meta (https://cran.r-project.org/web/packages/meta/index.html, version 4.18.1)^53^ and metafor (https://cran.r-project.org/web/packages/metafor/index.html, version 3.0.1)^54^ packages in R, version 4.03^55^. Since we have anticipated that the obtained studies will slightly differ in testing protocols and populations, we have opted for performing the meta-analysis using a random-effects model with inverse-variance method (the mathematical basis and motivating examples for this choice of model can be found in the paper by Borenstein et al.^56^). Statistically significant results have been defined as having p-value lesser than 0.05 for the null hypothesis that the effect size is 0 (with Knapp–Hartung adjustement^57^).To assess statistical heterogeneity Cochran’s Q test and I^2^ inconsistency statistic were calculated. To further explore heterogeneity, Baujat^58^ and graphical display of heterogeneity (GOSH)^59^ plots with three supervised machine learning (clustering) algorithms (k-means, DBSCAN, and Gaussian Mixture Model) were evaluated.

To compare effect sizes of surgical masks and N95 FFRs we have performed a network meta-analysis using the GeMTC package (https://cran.r-project.org/web/packages/gemtc/index.html, version 1.0.1)^52^. For each comparison simulations were repeated 20,000 with 5000 sample burn-in. Non-informative uniform prior distribution was used. Markov chain Monte Carlo simulations were performed to estimate posterior distributions. Convergence was assessed using trace plots and Brooks–Gelman–Rubin diagnostic statistics^60,61^. To assess inconsistency node-splitting analyses were performed^62^. Network geometry plots were constructed to visualize the evidence base. Line thickness corresponded to the proportion of participants in each comparison. The dotted line indicated indirect comparison.

Results from all meta-analyses were reported as standardized mean differences (SMD) with corresponding 95% confidence intervals (CI). Additionally, for network meta-analyses rank probabilities based on the surface under the cumulative ranking curve (SUCRA) were reported (probabilities that given intervention has greatest, second greatest, or least effect)^63^.

### Sensitivity analyses

We have performed sensitivity analyses with various values of imputed correlation coefficients for cross-over studies that did not report paired t-statistics. In addition, we have performed analyses with the exclusion of outlier studies and studies disproportionately contributing to heterogeneity, identified by visual inspection of Baujat and clustering of GOSH plots.

## Supplementary Information

Supplementary Information.
