# Supplementary material for: Bayesian network meta-analysis of face masks' impact on human physiology
Source: Sci Rep. 2022 Apr 6;12:5823. doi: 10.1038/s41598-022-09747-z (PMC8985742; doi:10.1038/s41598-022-09747-z)
Supplement: Supplementary file 1 — Supplementary Information. [file 41598_2022_9747_MOESM1_ESM.pdf]

Bayesian network meta-analysis of face masks' impact on human physiology

Kamil Litwinowicz*[1]

Marcin Choroszy[2]

Maciej Ornat[3]

Anna Wróbel[4]

Ewa Waszczuk[5]

[1] Department of Medical Biochemistry, Faculty of Medicine, Wroclaw Medical University

[2] Department of Microbiology, Wroclaw Medical University

[3] Department of Human Morphology and Embryology, Wroclaw Medical

[4] Department of Psychotherapy and Psychosomatic Diseases, Wroclaw Medical University

[5] Department of Gastroenterology and Hepatology, Wroclaw Medical University

Supplementary materials

Index

Search strategy……………………………………………………………………………………………1-3

Tables ………. 5

Risk of bias assessment…………………………………………………………………………………6

The geometry of comparisons…………………………………………………………………………7

Non-significant pairwise comparison ……………………………………………………………8-12

Funnel plot for pairwise comparisons ……………………………………………………………. 13-14

Heterogeneity plots………………………………………………………………………………………15 - 23

Sensitivity analyses……………………………………………………………………………………..24-31

Search strategy

- **WHO COVID DATABASE (https://search.bvsalud.org/global-literature-on-novel-coronavirus-2019-ncov/):**

(mask OR masks OR facemask* OR face-mask* OR MH:"Respiratory Protective Devices" OR N95 OR N97 OR N99 OR ffp OR ffp1 OR ffp2 OR ffp3 OR "respiratory protective supply" OR "respiratory protective equipment" OR "respiratory protective gear") AND (("tidal volume" OR "tidal volumes" OR "breathing frequency" OR "frequency of breathing" OR "respiratory rate" OR "respiratory rates" OR "partial pressure" OR "oxygen pressure" OR "heart rate" OR cardiopulmonary OR "carbon dioxide" OR saturation OR "blood pressure") OR ("body temperature" OR "skin temperature" OR "core temperature" OR "aural temperature" OR "exertion"))

- **MEDLINE (PUBMED, https://pubmed.ncbi.nlm.nih.gov/):**

((("surgical mask"[tw] OR "cloth mask"[tw] OR "surgical masks"[tw] OR "cloth masks"[tw] OR "medical mask"[tw] OR "medical masks" OR "Respiratory Protective Devices"[MeSH Terms] OR N95[tw] OR N97[tw] OR N99[tw] OR ffp[tw] OR ffp1[tw] OR ffp2[tw] OR ffp3[tw] OR "respiratory protective equipment"[tw] OR respiratory protective gear[tw] OR facemask*[tw] OR face-mask*[tw]) OR ((mask[tw] OR masks[tw]) AND ("Protective Devices"[MeSH Terms] OR "Infection control"[MeSH terms]))) AND (tidal volume*[tw] OR breathing frequency[tw] OR frequency of breathing[tw] OR respiratory rate*[tw] OR partial pressure[tw] OR oxygen pressure[tw] OR heart rate[tw] OR cardiopulmonary[tw] OR carbon dioxide OR saturation[tw] OR "blood pressure"[tw] OR "body temperature"[tw] OR "skin temperature"[tw] OR "core temperature" OR "aural temperature" OR "exertion")) NOT (CPAP[tw] OR "positive airway pressure"[tw] OR "laryngeal mask*"[tw] OR anaesthesia OR postoperative[tw] OR "Perioperative Period"[MeSH Terms] OR "noninvasive ventilation" OR "non-invasive ventilation" OR "mask ventilation" OR preoxygenation OR pre-oxygenation OR "Respiratory Therapy"[MeSH Terms] OR (animal[tw] NOT (animal[tw] AND human[tw])))

- **CINAHL (EBSCOhost, https://search.ebscohost.com/):**

((("surgical mask" OR "cloth mask" OR "surgical masks" OR "cloth masks" OR "medical mask" OR "medical masks"

OR (MH "Respiratory Protective Devices+") OR N95 OR N97 OR N99 OR ffp OR ffp1 OR ffp2 OR ffp3 OR "respiratory protective equipment" OR "respiratory protective gear" OR facemask* OR face-mask*) OR ((mask OR masks) AND ((MH "Protective Devices+") OR (MH "Infection control+")))) AND ("tidal volume*" OR "breathing frequency" OR "frequency of breathing" OR "respiratory rate*" OR "partial pressure" OR "oxygen pressure" OR "heart rate" OR cardiopulmonary OR "carbon dioxide" OR "blood pressure"

OR saturation OR "body temperature" OR "skin temperature" OR "core temperature" OR "aural temperature" OR "exertion")) NOT (CPAP OR "positive airway pressure" OR "laryngeal mask*" OR anaesthesia

OR postoperative OR (MH "Perioperative Period+") OR "noninvasive ventilation"

OR "non-invasive ventilation"

OR "mask ventilation"

OR preoxygenation

OR pre-oxygenation

OR (MH "Respiratory Therapy+") OR (animal NOT (animal AND human)))

- **CENTRAL (https://www.cochranelibrary.com/central/):**

ID Search

#1 "surgical mask" OR "cloth mask" OR "surgical masks" OR "cloth masks" OR "medical mask" OR "medical masks" OR "N95" OR "N97" OR "N99" OR ffp OR ffp1 OR ffp2 OR ffp3 OR "respiratory protective equipment" OR "respiratory protective gear" OR facemask* OR face-mask*

#2 MeSH descriptor: [Protective Devices] explode all trees

#3 MeSH descriptor: [Infection Control] explode all trees

#4 (mask OR masks) AND (#2 OR #3)

#5 #1 OR #4

#6 "tidal volume*" OR "breathing frequency" OR "frequency of breathing" OR "respiratory rate*" OR "partial pressure" OR "oxygen pressure" OR "heart rate" OR cardiopulmonary OR "carbon dioxide" OR "blood pressure” OR saturation OR "body temperature" OR "skin temperature" OR "core temperature" OR "aural temperature" OR "exertion”

#7 #5 AND #6

#8 MeSH descriptor: [Perioperative Care] explode all trees

#9 CPAP OR "positive airway pressure" OR "laryngeal mask*" OR anaesthesia

OR postoperative OR "noninvasive ventilation"

OR "non-invasive ventilation"

OR "mask ventilation"

OR preoxygenation

OR pre-oxygenation

#10 (animal NOT (animal AND human))

#11 #7 NOT (#8 OR #9 OR #10)

- **Embase (https://www.embase.com/):**

((("surgical mask":ti,ab,de,tn OR "cloth mask":ti,ab,de,tn OR "surgical masks":ti,ab,de,tn OR "cloth masks":ti,ab,de,tn OR "medical mask":ti,ab,de,tn OR "medical masks"

OR 'Respiratory Protective Devices'/exp OR N95 OR N97 OR N99 OR ffp:ti,ab,de,tn OR ffp1:ti,ab,de,tn OR ffp2:ti,ab,de,tn OR ffp3:ti,ab,de,tn OR "respiratory protective equipment":ti,ab,de,tn OR "respiratory protective gear":ti,ab,de,tn OR facemask*:ti,ab,de,tn OR face-mask*:ti,ab,de,tn) OR ((mask:ti,ab,de,tn OR masks:ti,ab,de,tn) AND ('Protective Devices'/exp OR 'Infection control'/exp))) AND ("tidal volume*":ti,ab,de,tn OR "breathing frequency":ti,ab,de,tn OR "frequency of breathing":ti,ab,de,tn OR "respiratory rate*":ti,ab,de,tn OR "partial

pressure":ti,ab,de,tn OR "oxygen pressure":ti,ab,de,tn OR "heart rate":ti,ab,de,tn OR cardiopulmonary:ti,ab,de,tn OR "carbon dioxide"

OR saturation:ti,ab,de,tn OR "blood pressure":ti,ab,de,tn OR "body temperature":ti,ab,de,tn OR "skin temperature":ti,ab,de,tn OR "core temperature"

OR "aural temperature"

OR exertion

)) NOT (CPAP:ti,ab,de,tn OR "positive airway pressure":ti,ab,de,tn OR "laryngeal mask*":ti,ab,de,tn OR anaesthesia

OR postoperative:ti,ab,de,tn OR 'Perioperative Period'/exp OR "noninvasive ventilation"

OR "non-invasive ventilation"

OR "mask ventilation"

OR preoxygenation

OR pre-oxygenation

OR 'Respiratory Therapy'/exp OR (animal:ti,ab,de,tn NOT (animal:ti,ab,de,tn AND human:ti,ab,de,tn)))

TABLES

	Carrizal et al.
Is the case definition adequate?	0
Representativeness of the cases	1
Selection of Controls	1
Definition of Controls	1
Comparability of cases and controls on the basis of the design or analysis	2
Ascertainment of exposure	1
Same method of ascertainment for cases and controls	1
Non-Response rate	1

Supplementary Table 1. Risk of bias assessment of non-randomized studies

Risk of bias assessment

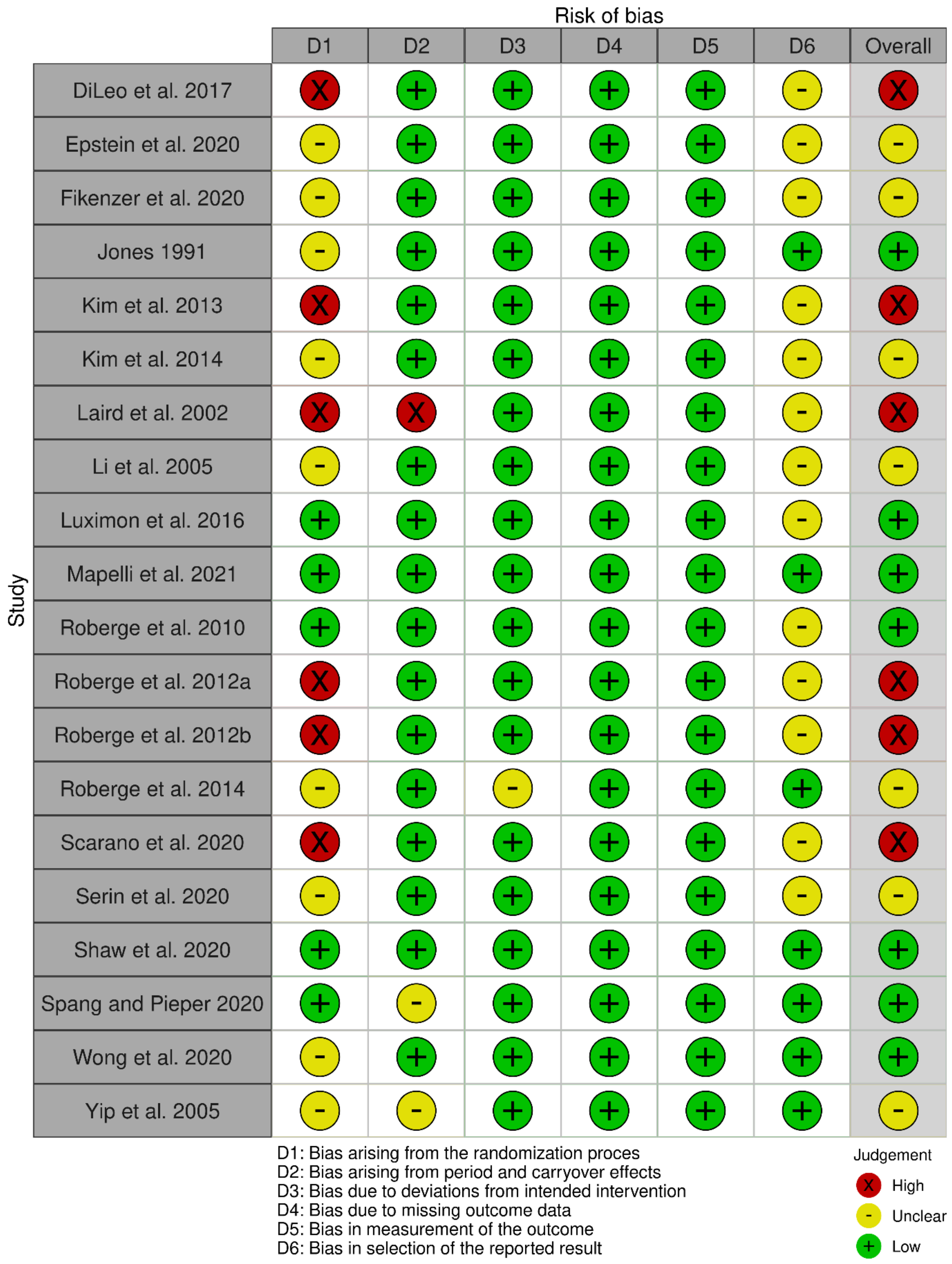

Supplementary Fig. 1 “Traffic light” plot of risk of bias assessment (based on the second version of Cochrane risk of bias tool).

The geometry of comparisons

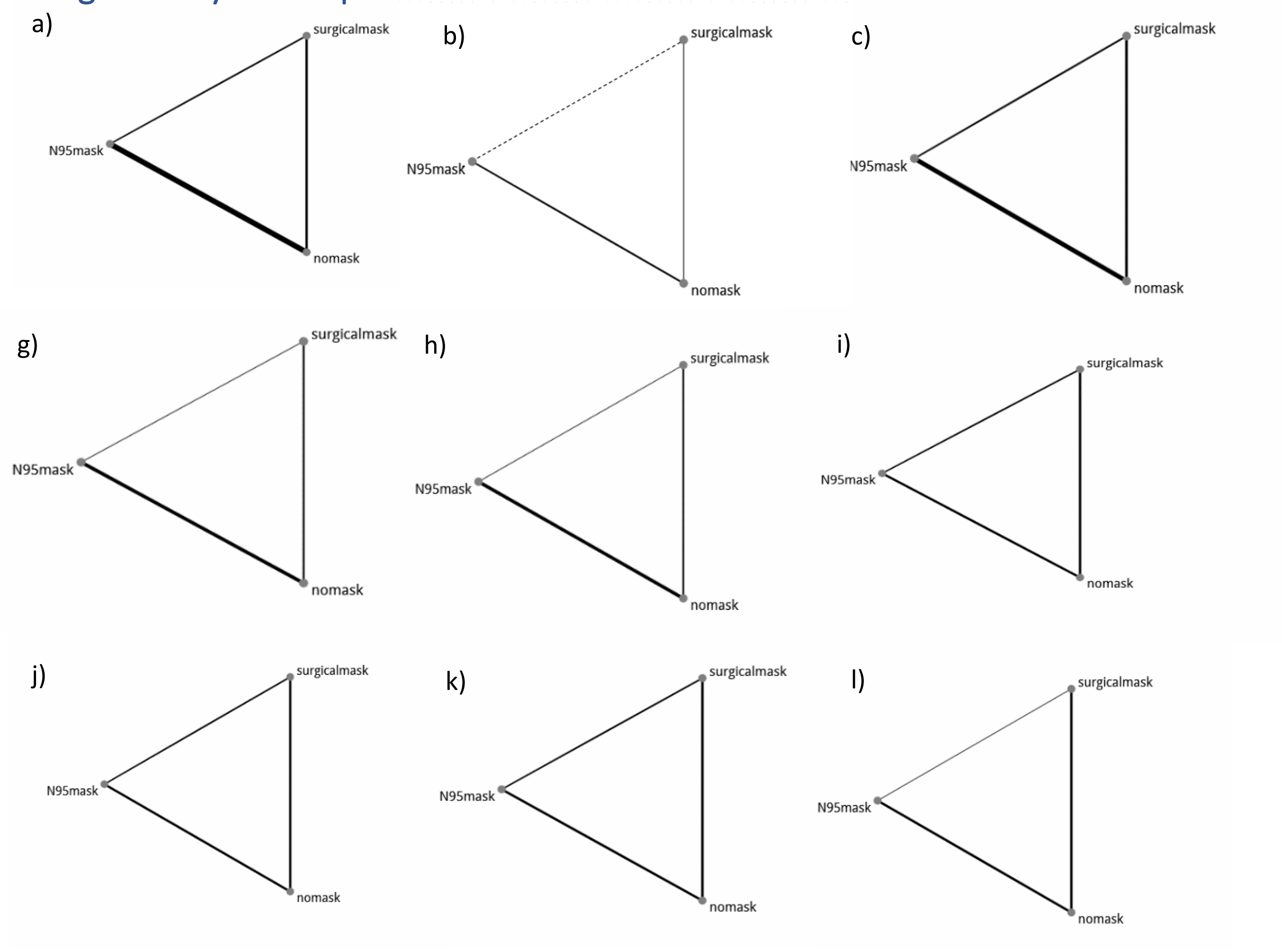

Supplementary Fig. 2. The geometry of the comparisons; a. Temperature of covered facial skin, b. RHP, c. RR, d. The temperature of facial skin not covered by a mask, e. Aural temperature, f. HR, g. SpO2, h. tcPCO2, i. HR during high-intensity exercise, j. HR during moderate-intensity exercise, k. SBP, l. RPE

a) Aural temperature, N95 FFR

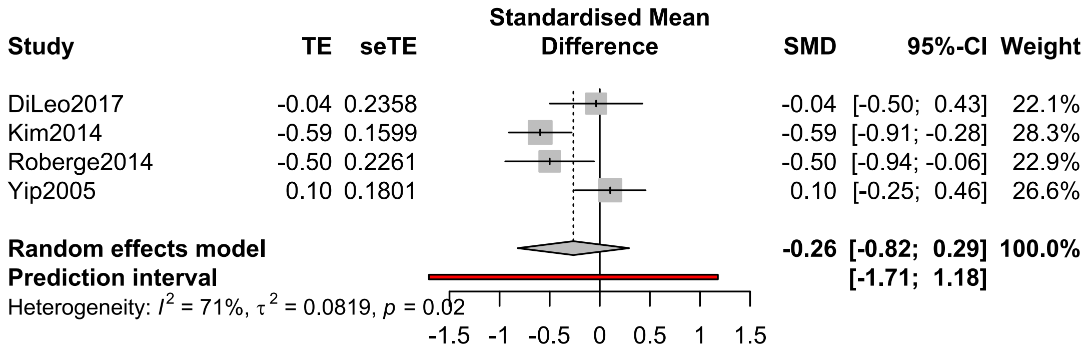

b) Covered facial skin temperature, surgical mask

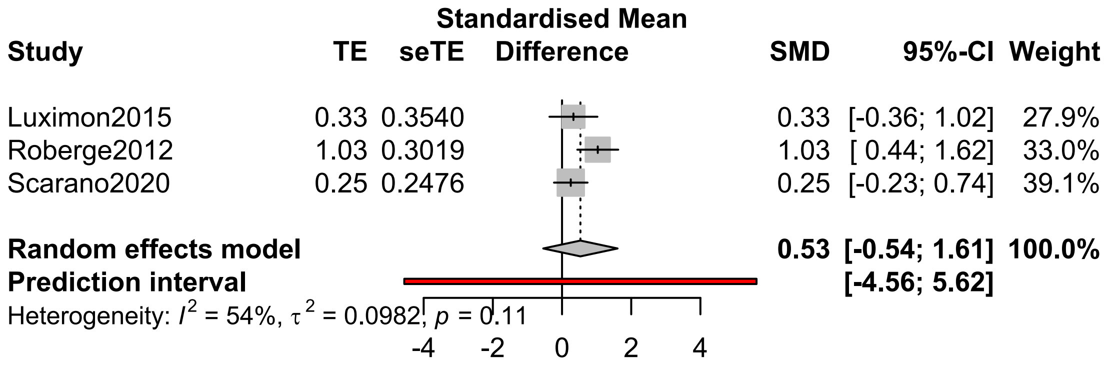

c) HR during high intensity exercise, N95 FFR

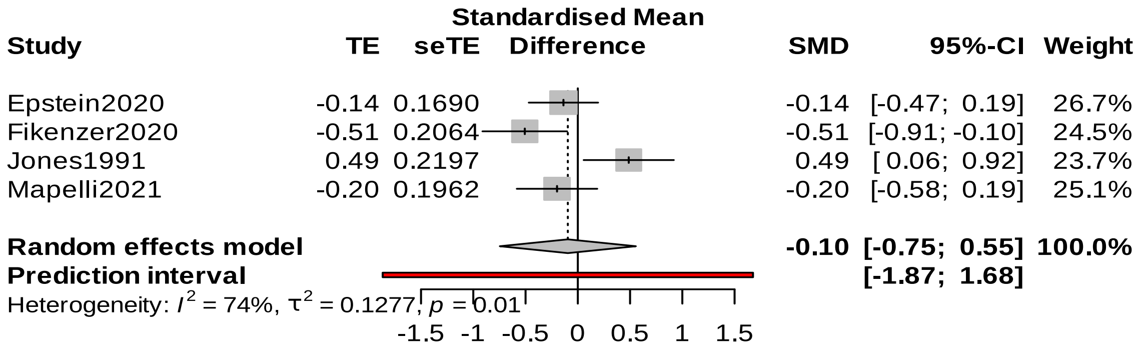

d) HR during high intensity exercise, surgical mask

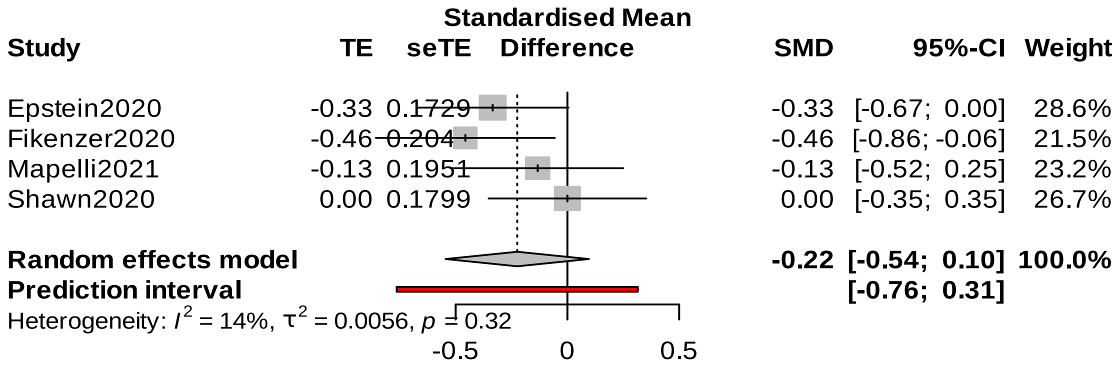

e) HR during moderate intensity exercise, N95 FFR

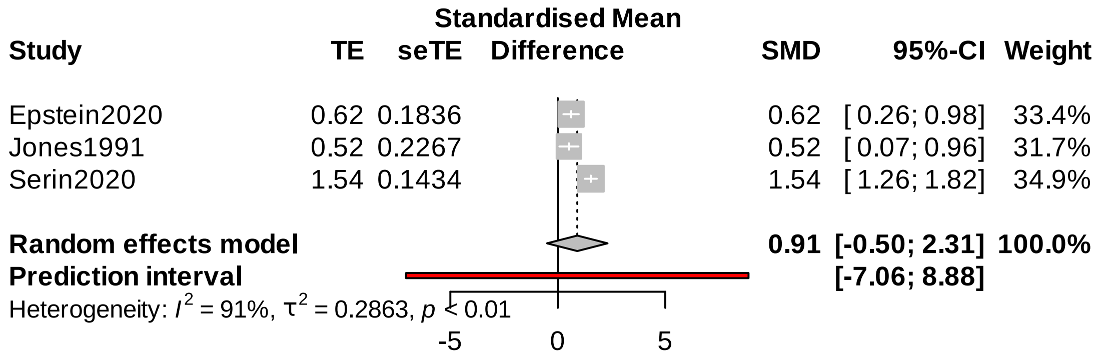

f) HR during moderate intensity exercise, surgical mask

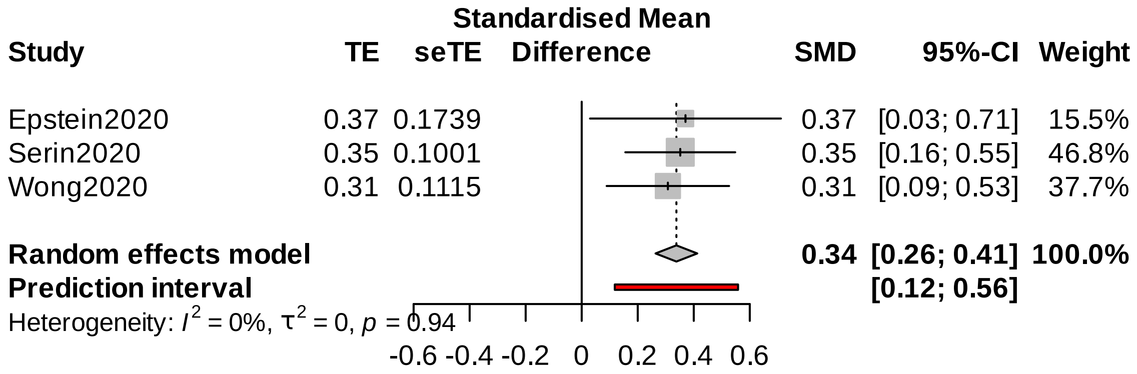

g) HR, surgical mask

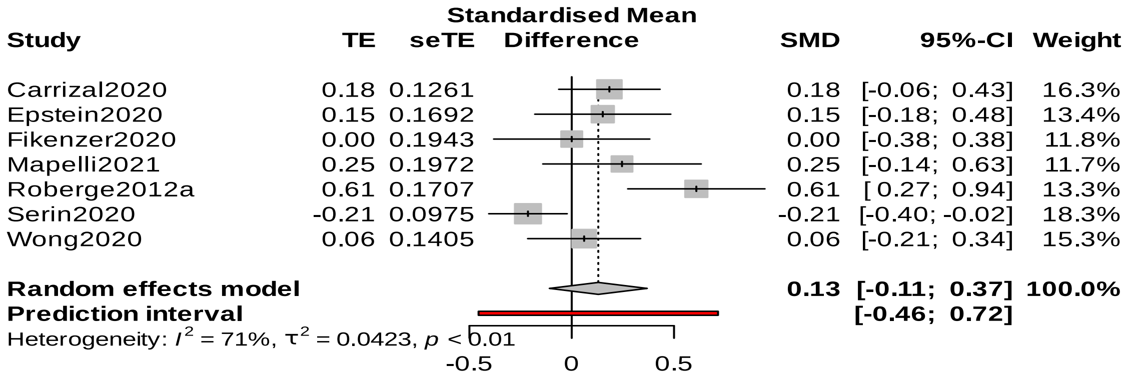

h) RHP, surgical mask

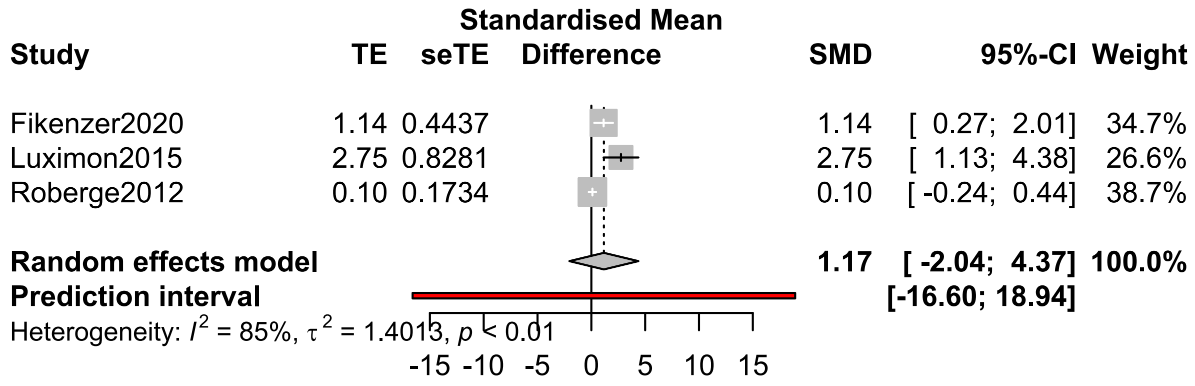

i) RPE, N95 FFR

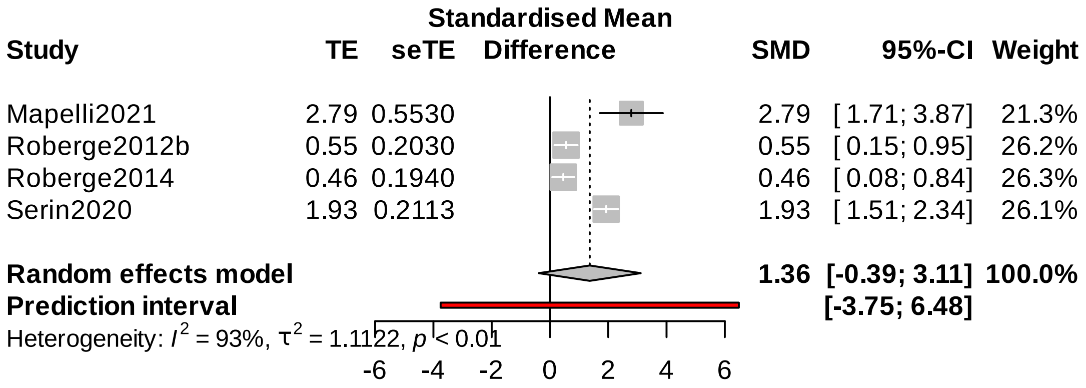

j) RPE, surgical mask

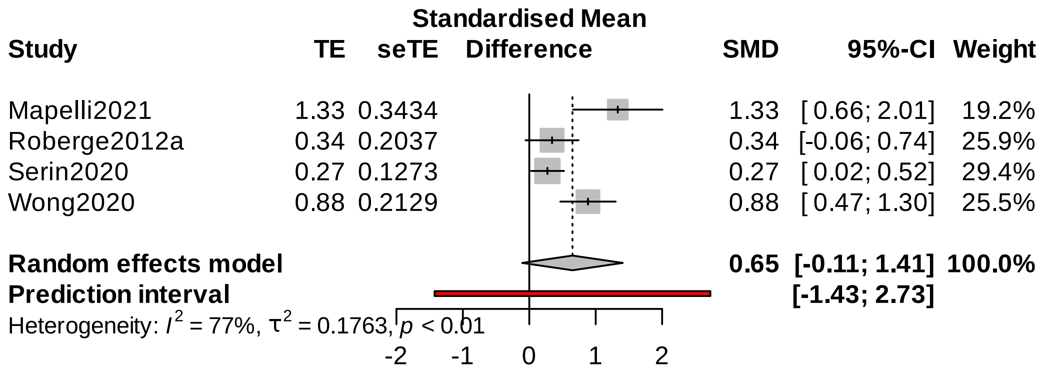

k) RR, N95 FFR

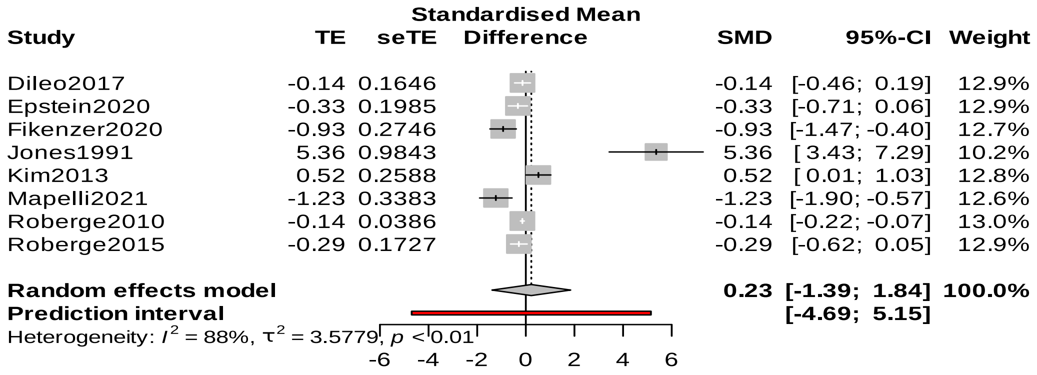

l)RR, surgical mask

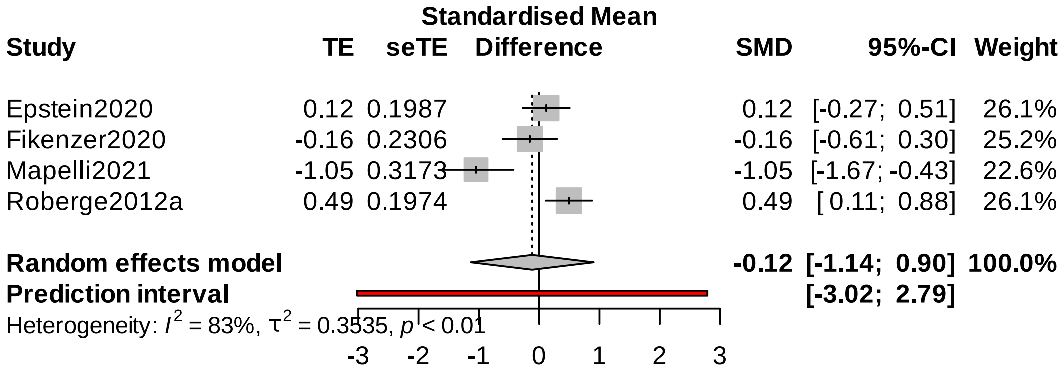

m) SBP, N95 FFR

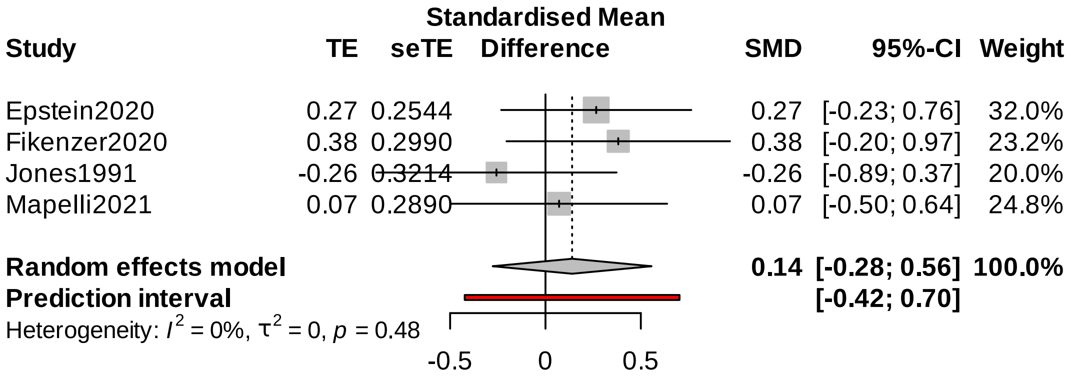

n) SBP, surgical mask

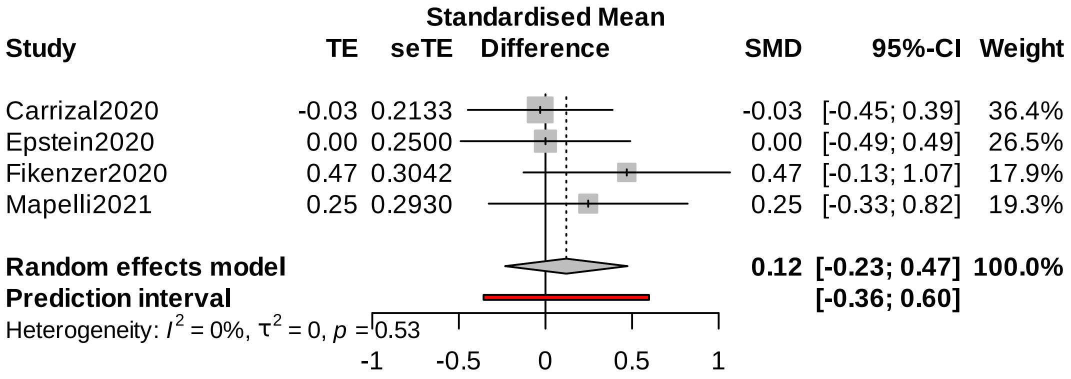

o) SpO2, N95 FFR

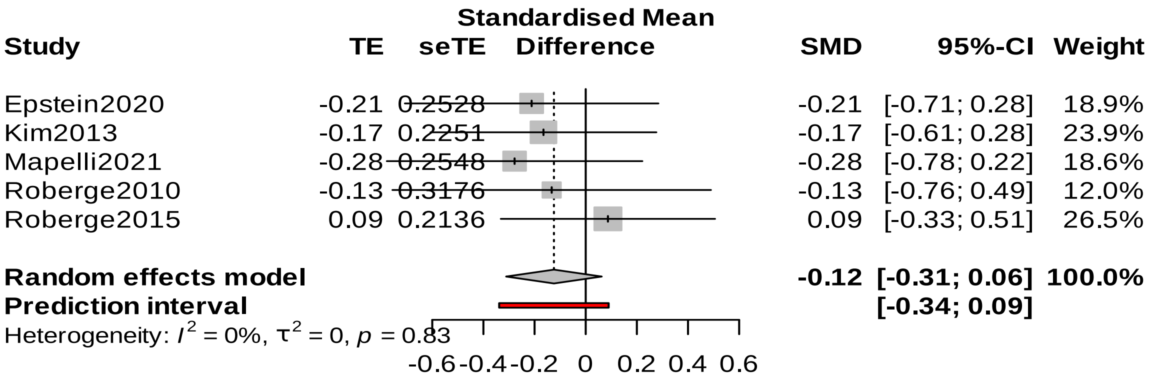

p) Uncovered facial skin temperature, N95 FFR

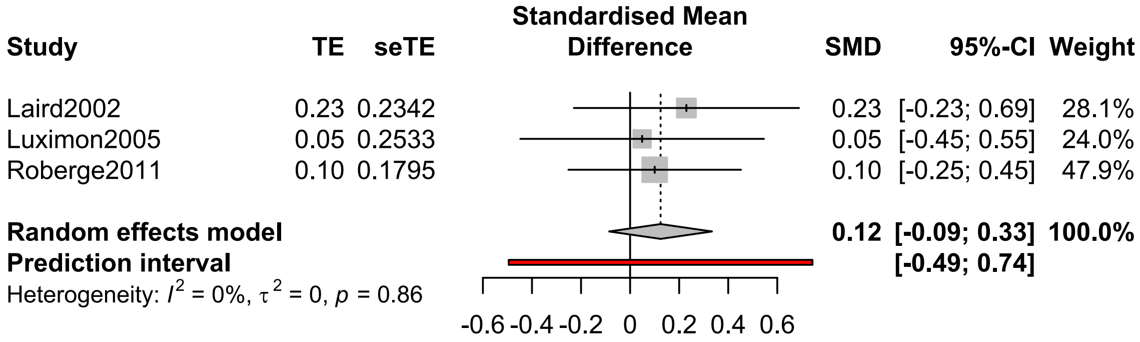

r) SpO22 during high intensity conditio, surgical mask

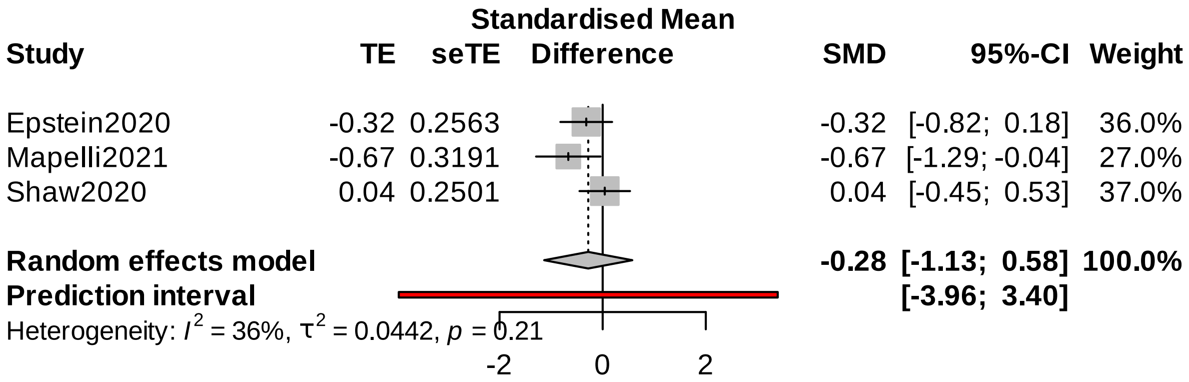

s) tidal volume, N95 FFR

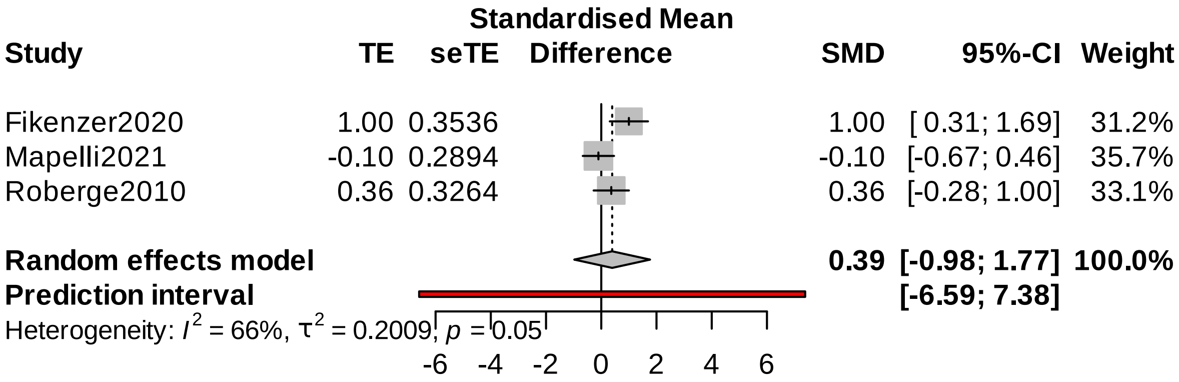

Supplementary Fig. 3. Non-significant pairwise comparison

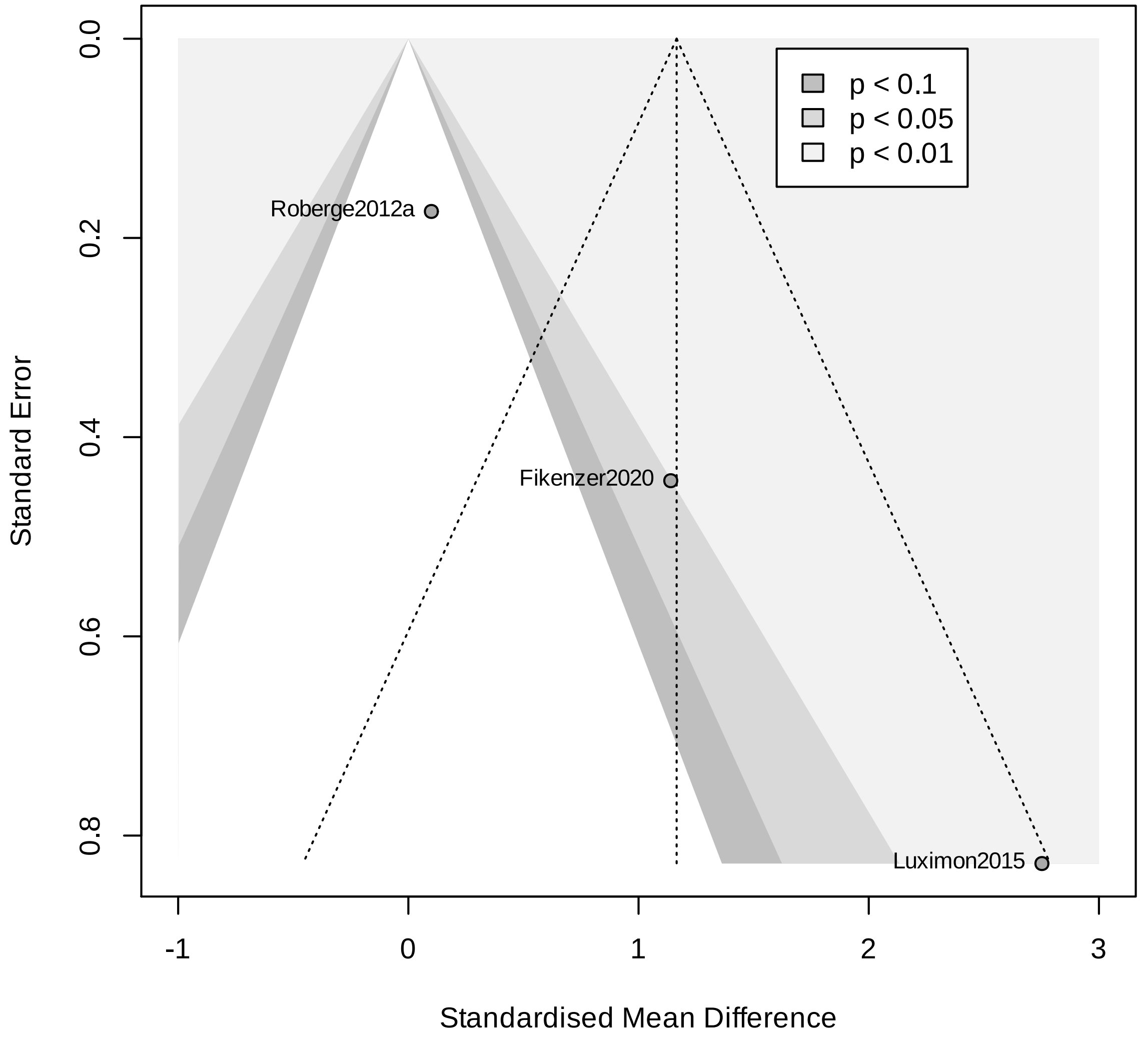

Supplementary Fig. 4. Funnel plot for pairwise comparison on RHP, surgical mask

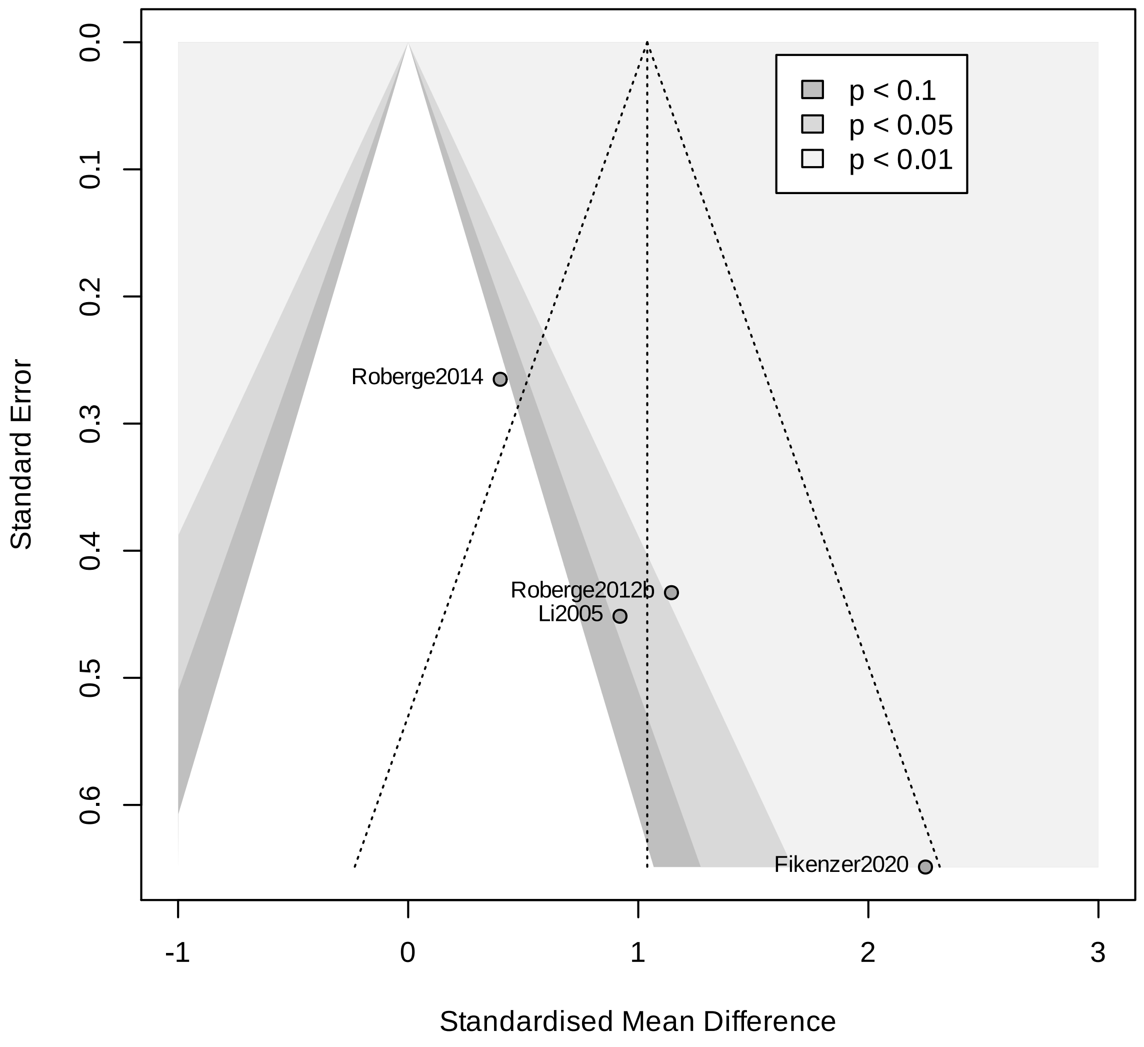

Supplementary Fig. 5. Funnel plot for pairwise comparison on RHP, N95 mask

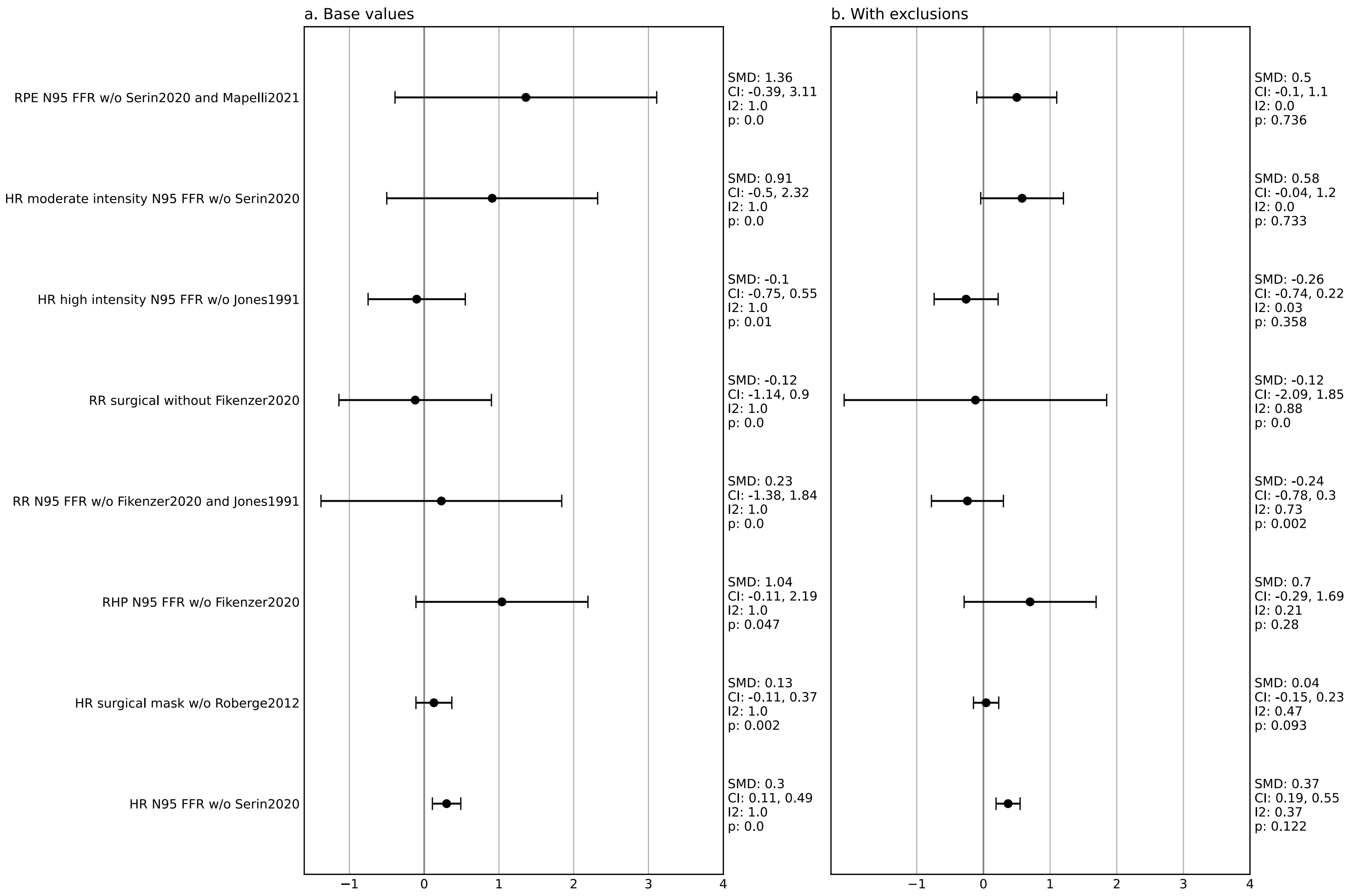

Supplementary Fig. 6. Sensitivity analysis with exclusion of studies overly contributing to heterogeneity; p - p-value for test of heterogeneity, I2 – Higgins and Thompson I^2 statistic

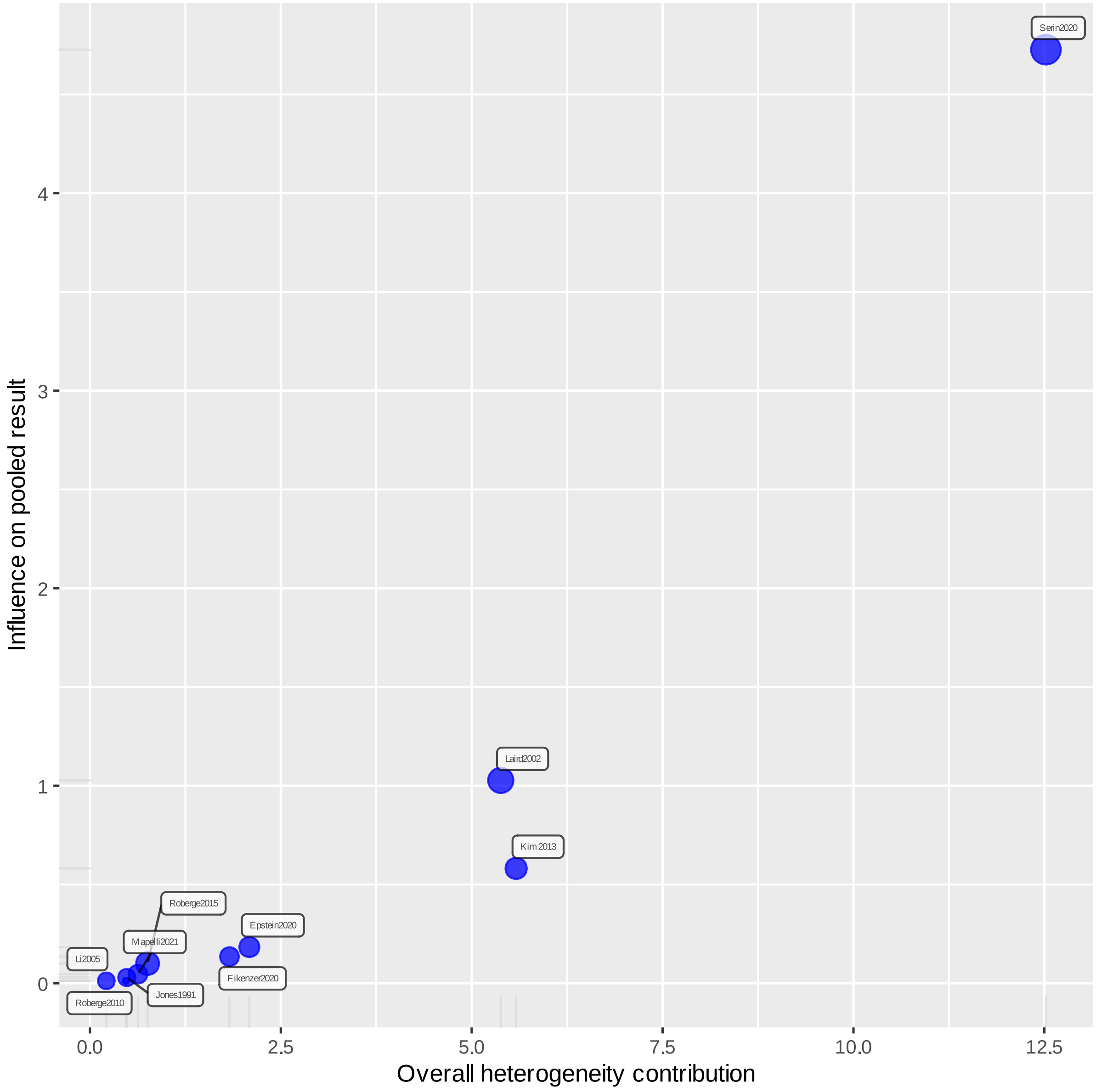

Supplementary Fig. 7. Visual representation of heterogeneity in pairwise comparison of N95 FFR's effect on heart rate.

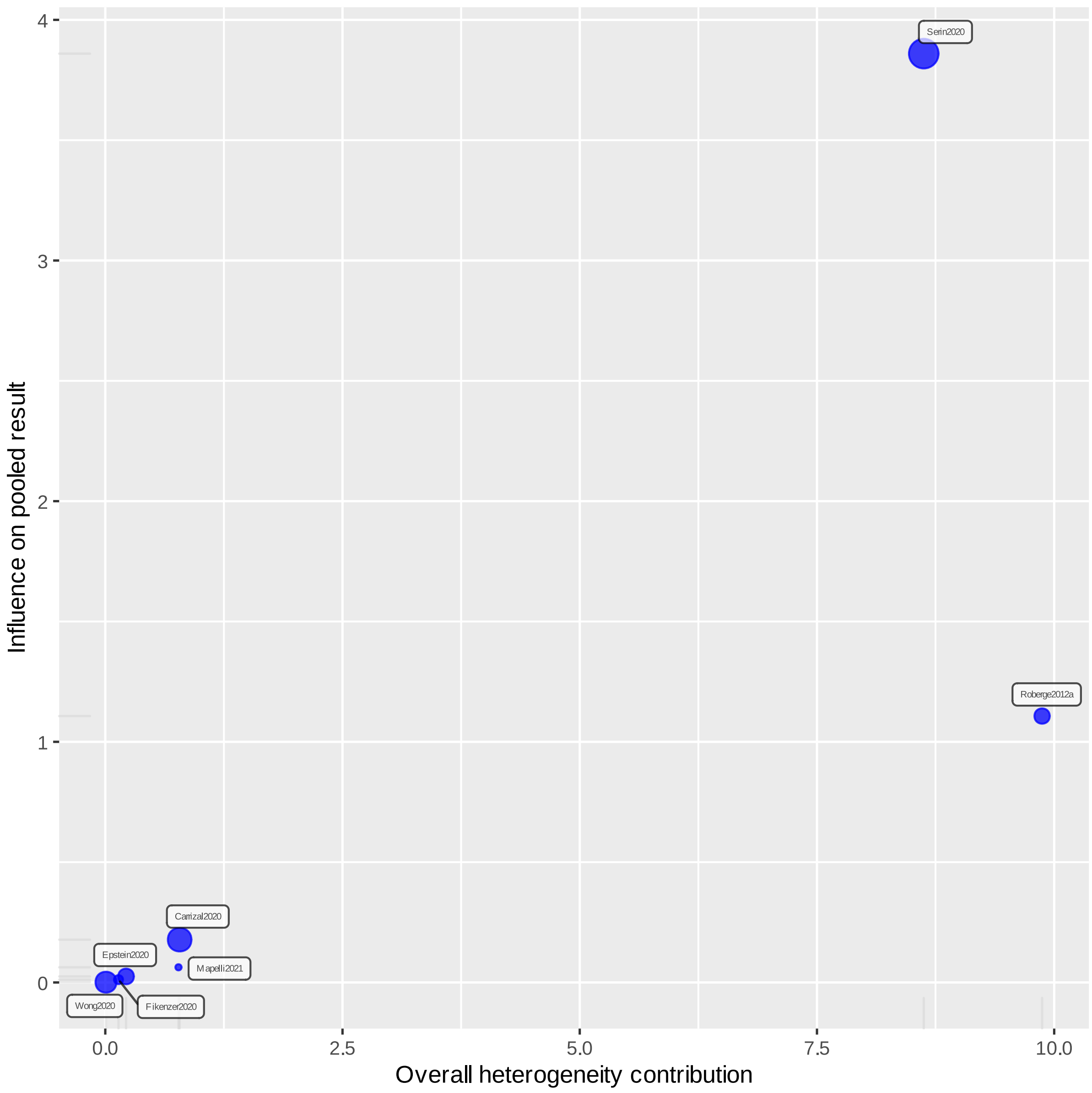

Supplementary Fig. 8. Visual representation of heterogeneity in pairwise comparison of surgical mask's effect on heart rate.

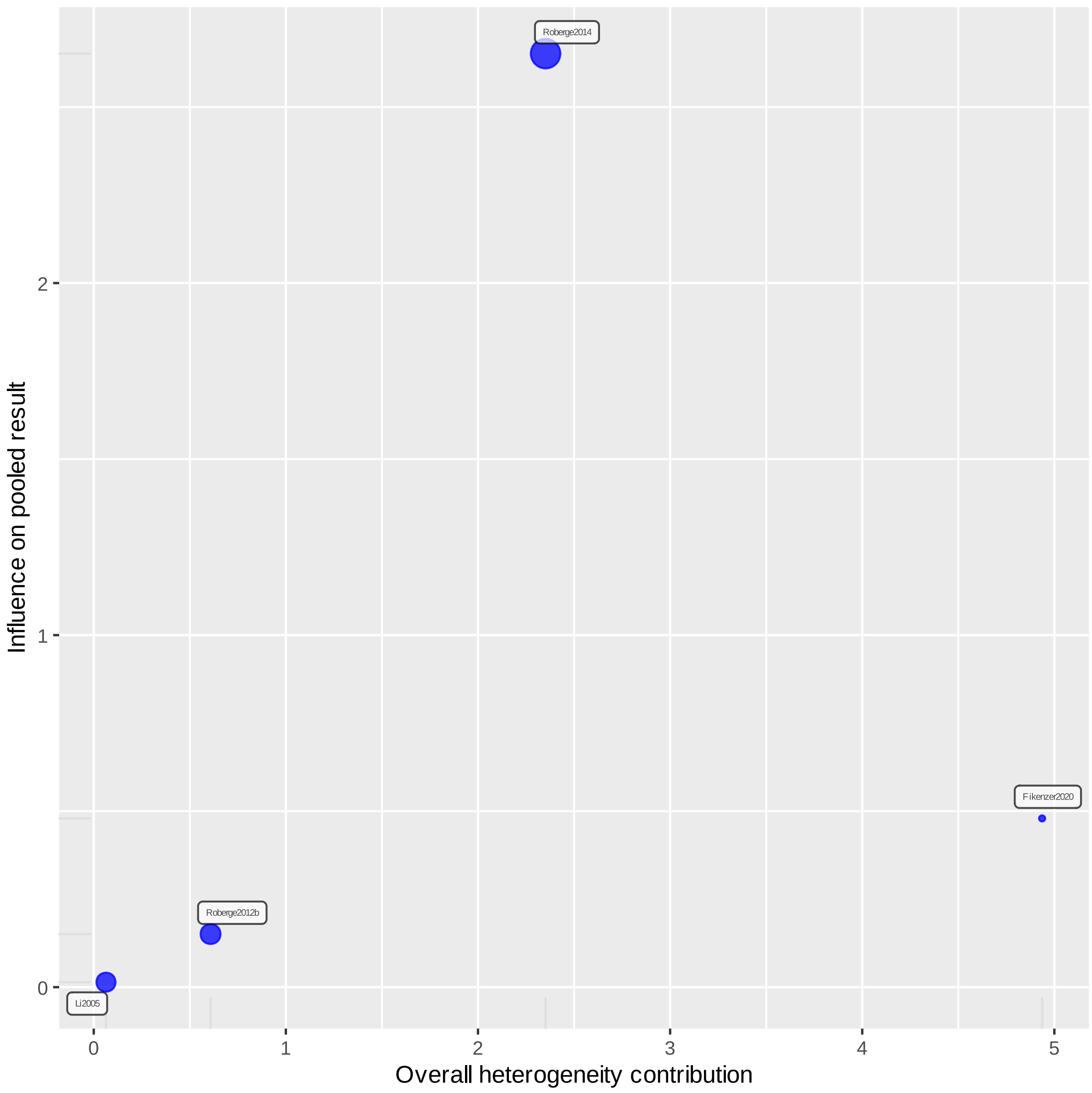

Supplementary Fig. 9. Visual representation of heterogeneity in pairwise comparison of N95 FFR's effect on rating of heat perception.

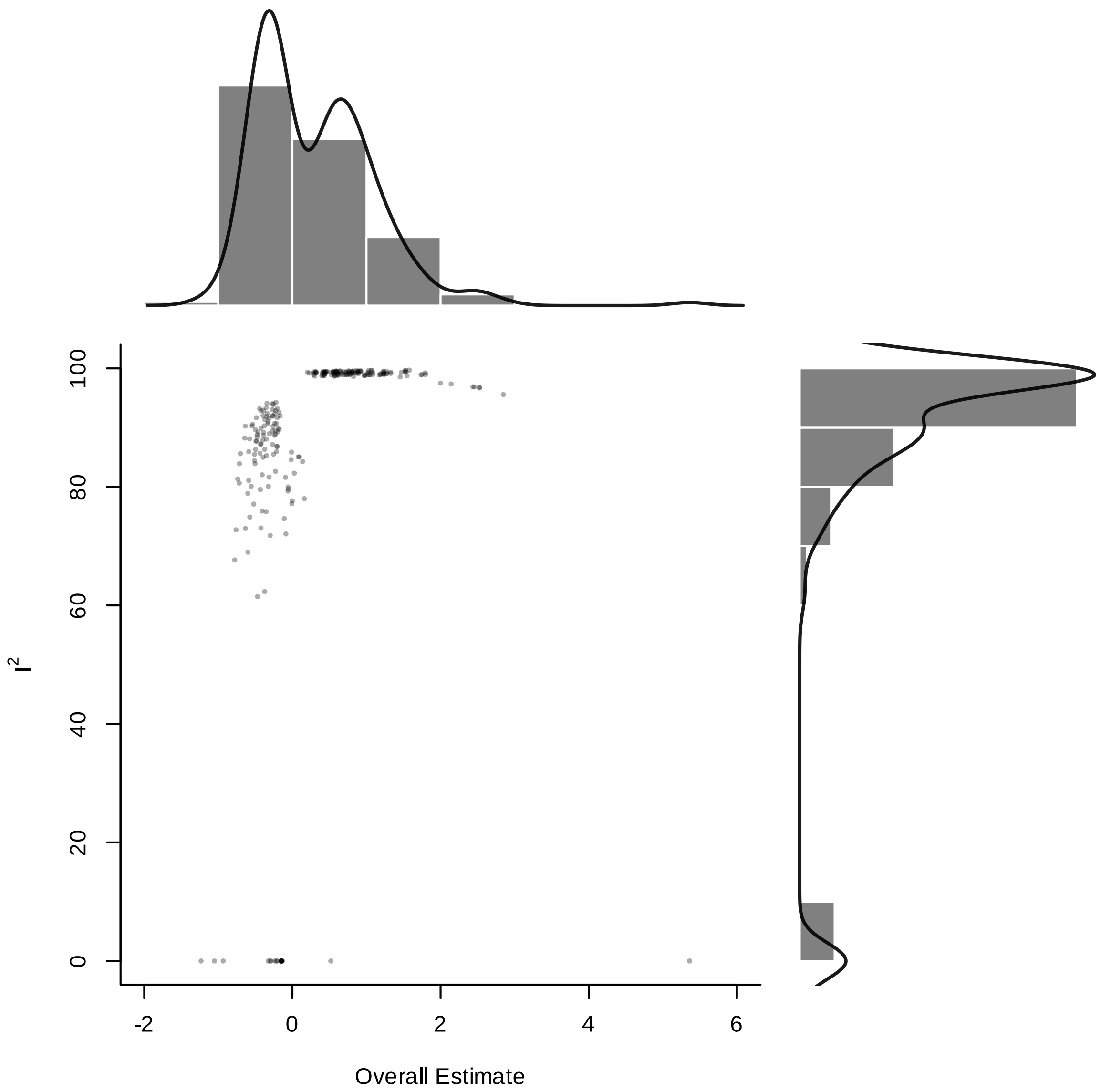

Supplementary Fig. 10. GOSH plot of N95 FFR's effect on respiratory rate. Clustering algorithms revealed that study by Fikenzer et al. (2020) and Jones et al. (1991) was overly contributing to heterogeneity.

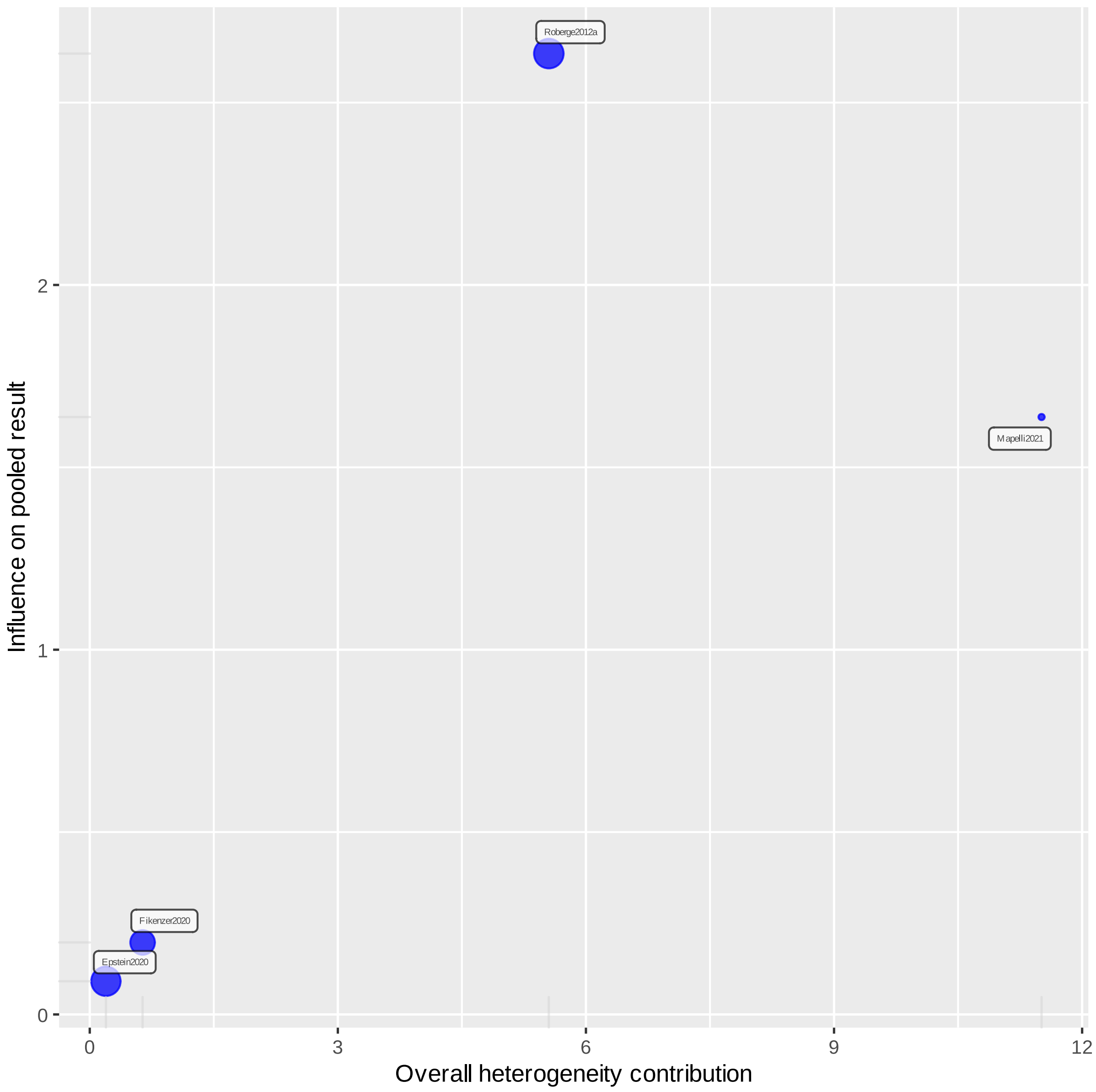

Supplementary Fig. 11. Visual representation of heterogeneity in pairwise comparison of surgical mask's effect on respiratory rate.

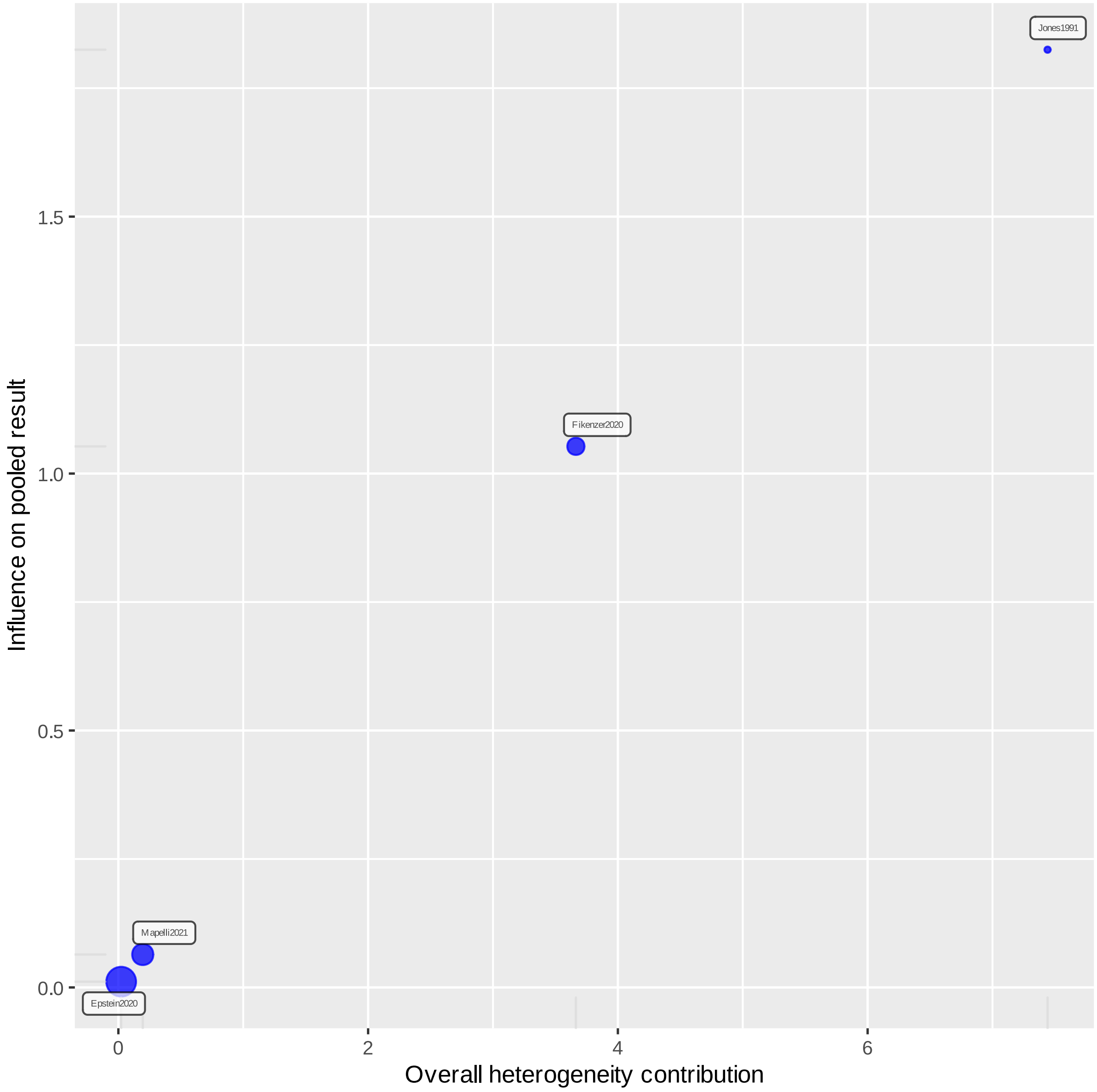

Supplementary Fig. 12. Visual representation of heterogeneity in pairwise comparison of N95 FFR's effect on heart rate during high intensity activity.

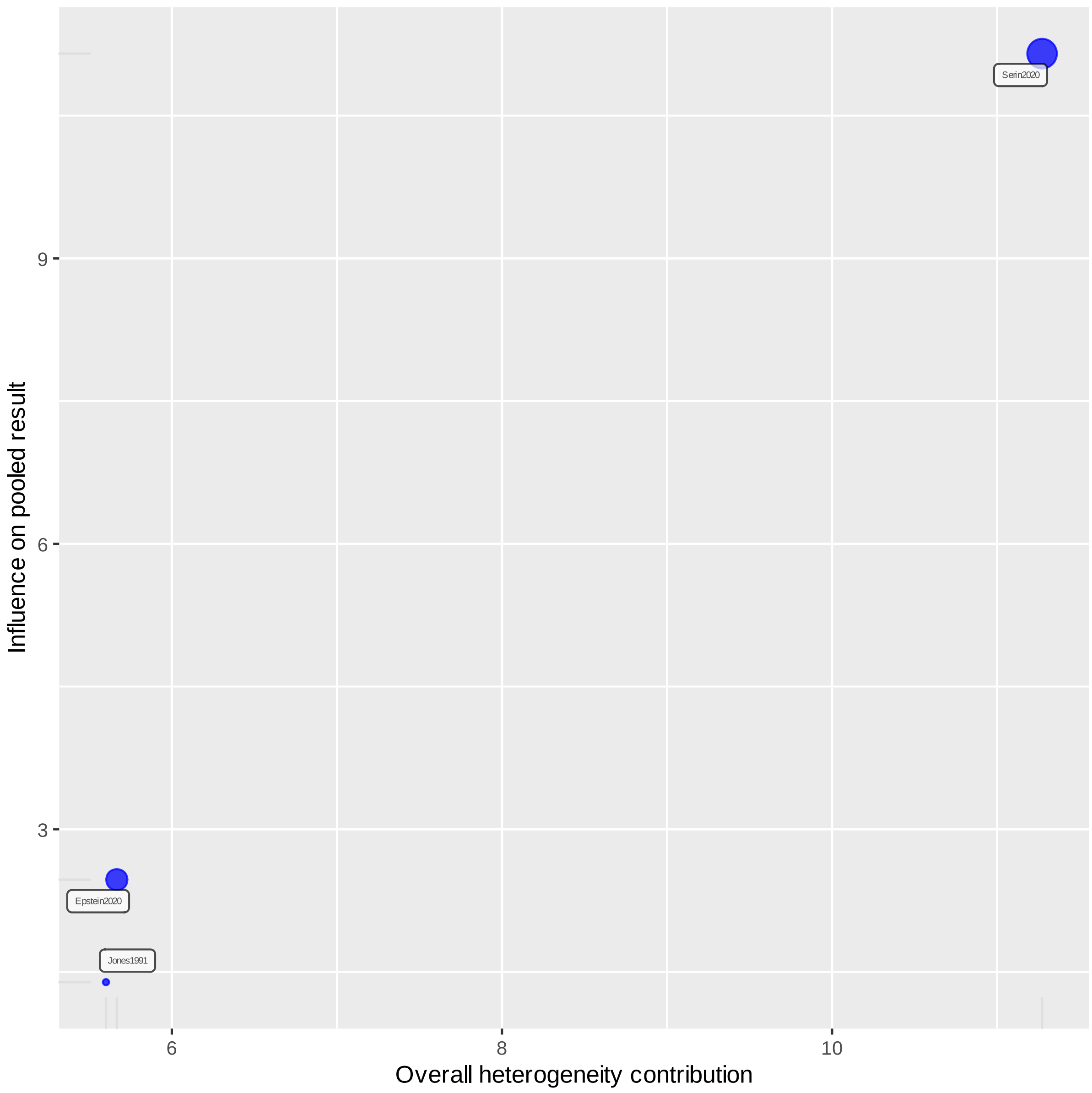

Supplementary Fig. 13. Visual representation of heterogeneity in pairwise comparison of N95 FFR's effect on heart rate during moderate intensity activity.

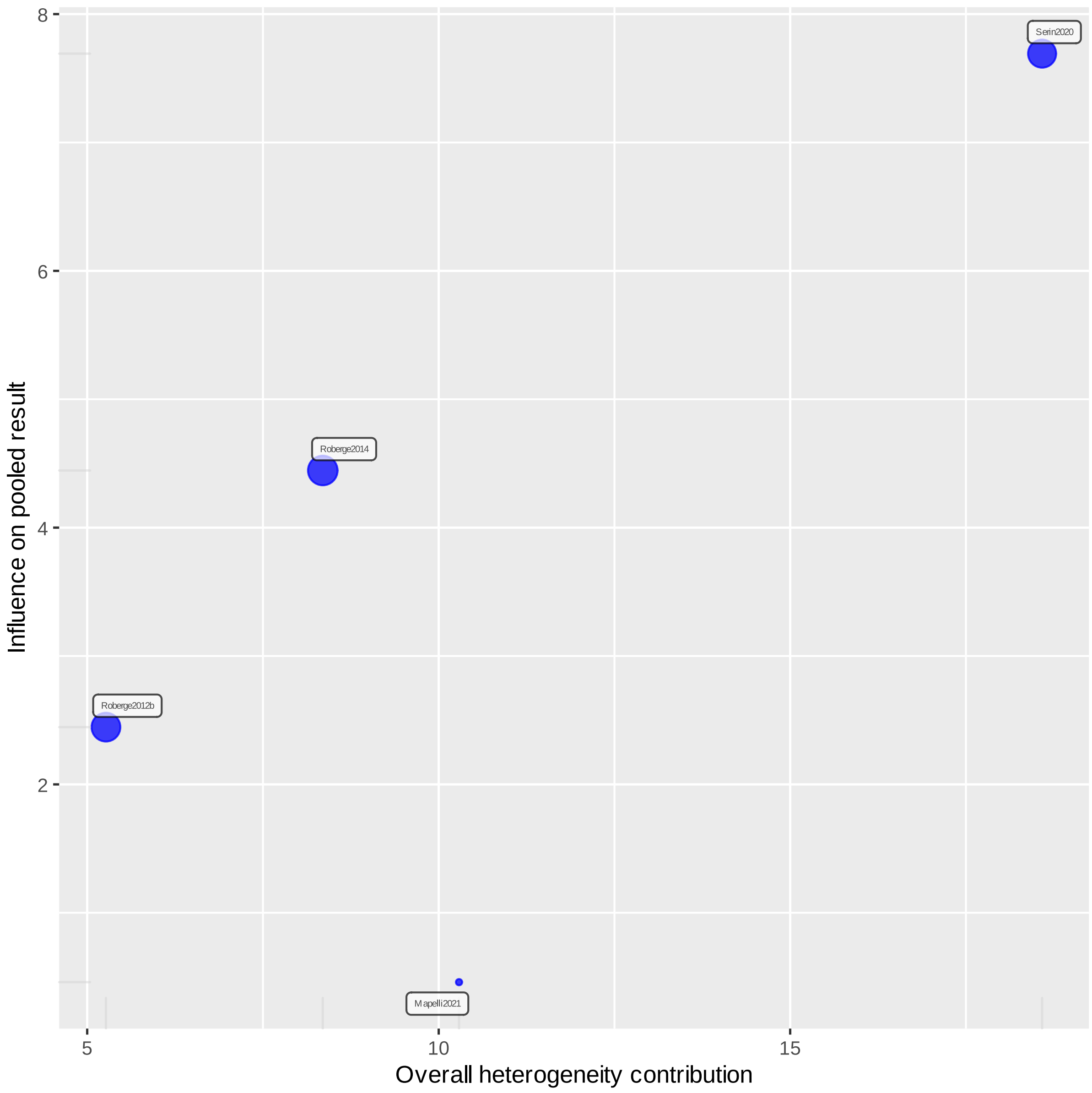

Supplementary Fig. 14. Visual representation of heterogeneity in pairwise comparison of N95 FFR's effect on rating of perceived exertion

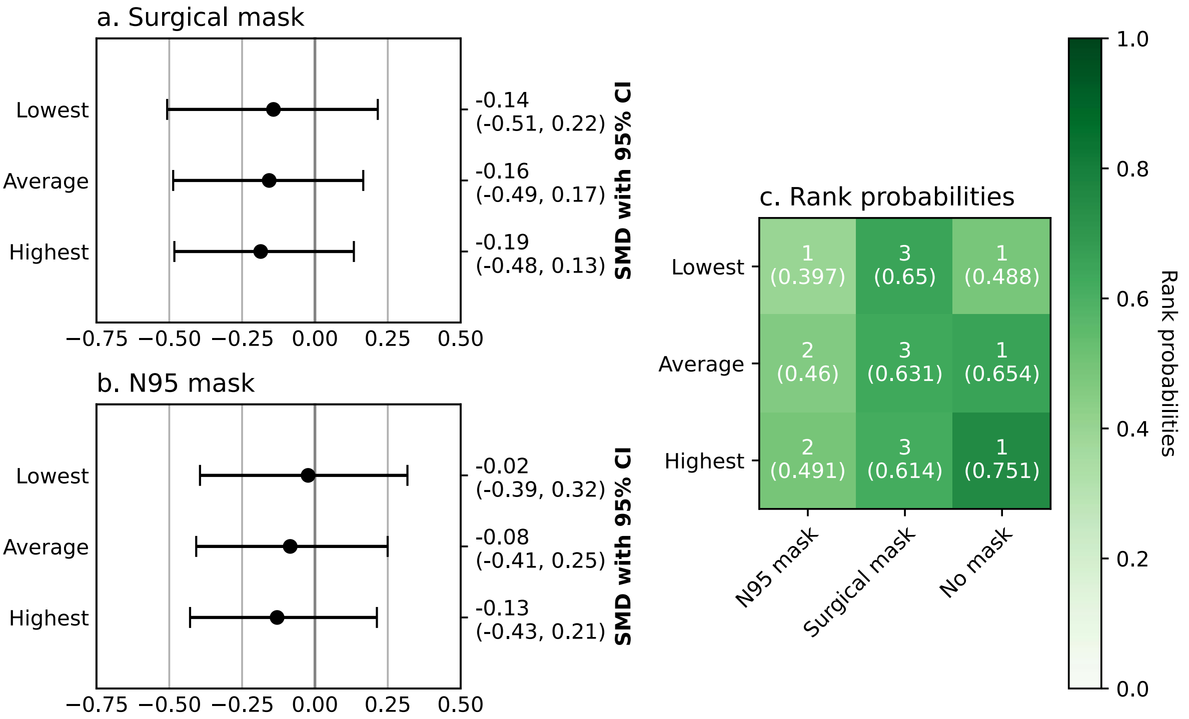

Supplementary Fig. 15. Sensitivity analysis of various imputed correlation coefficients for HR during high-intensity exercise. Numbers in rank probabilities heatmap represent rank with the highest probability and corresponding probability for given measure and condition

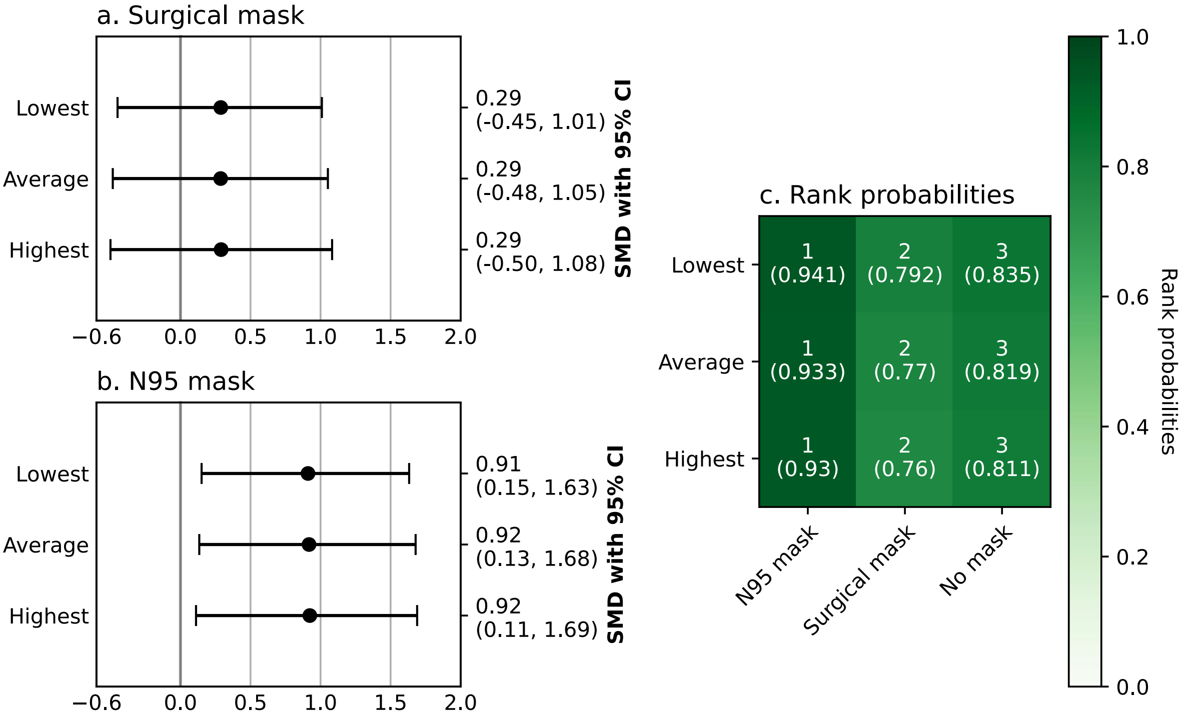

Supplementary Fig. 16. Sensitivity analysis of various imputed correlation coefficients for HR during moderate-intensity exercise. Numbers in rank probabilities heatmap represent rank with the highest probability and corresponding probability for given measure and condition

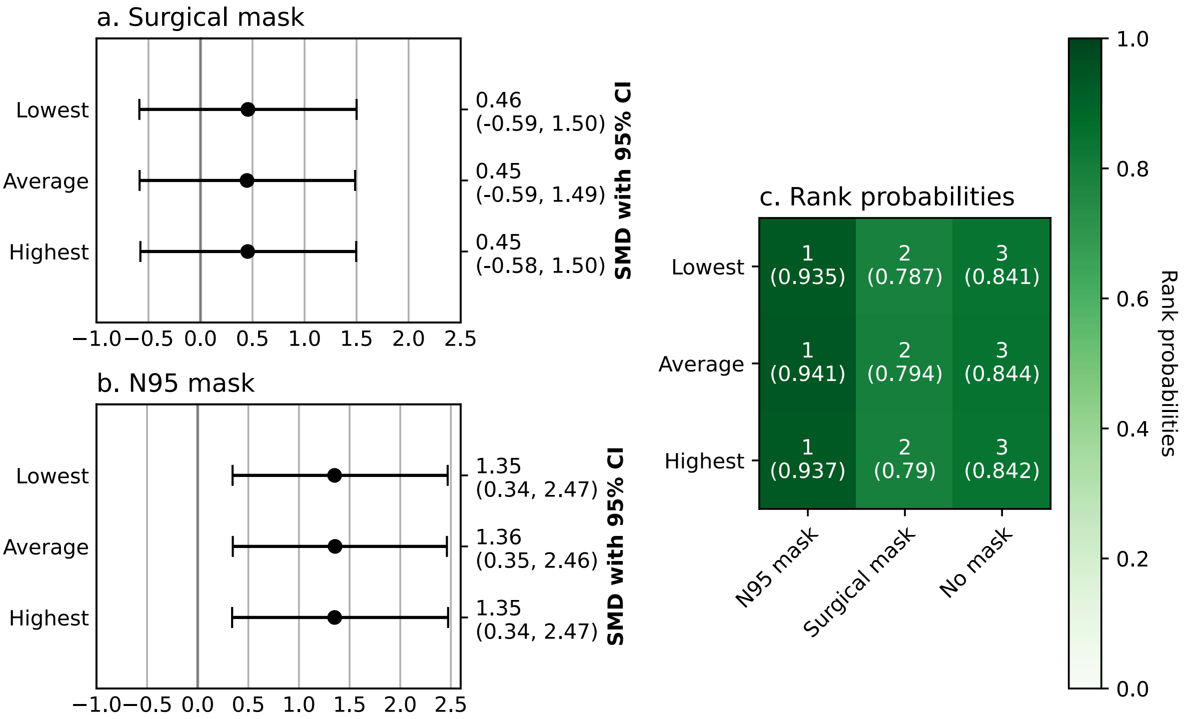

Supplementary Fig. 17. Sensitivity analysis of various imputed correlation coefficients for RPE. Numbers in rank probabilities heatmap represent rank with the highest probability and corresponding probability for given measure and condition

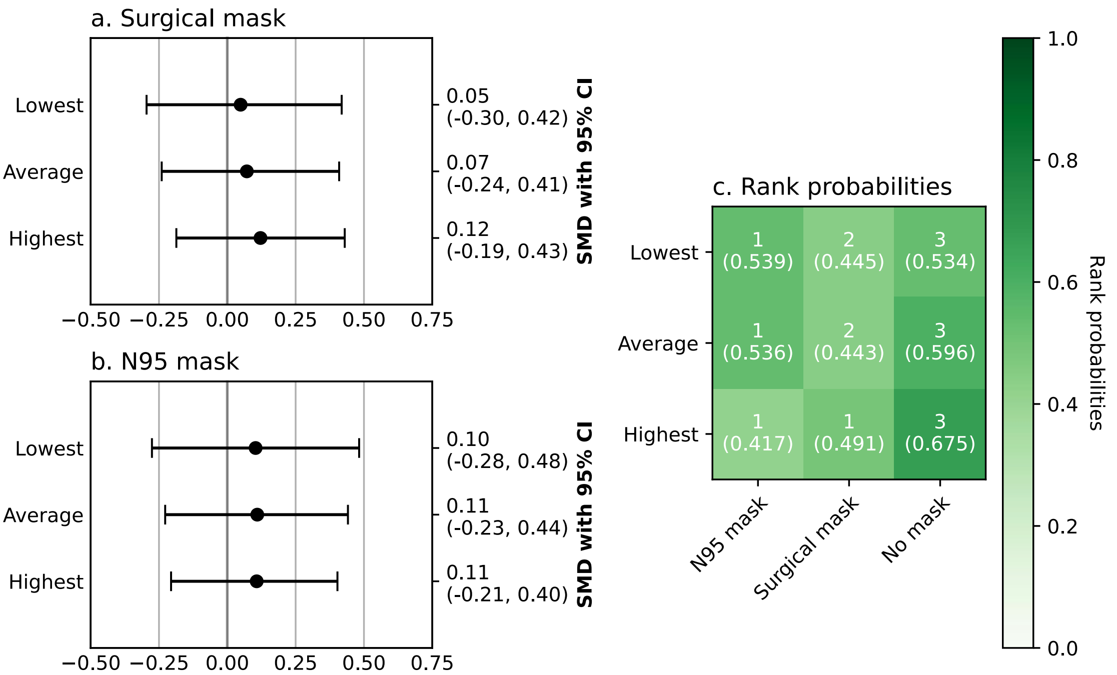

Supplementary Fig. 18. Sensitivity analysis of various imputed correlation coefficients for SBP. Numbers in rank probabilities heatmap represent rank with the highest probability and corresponding probability for given measure and condition

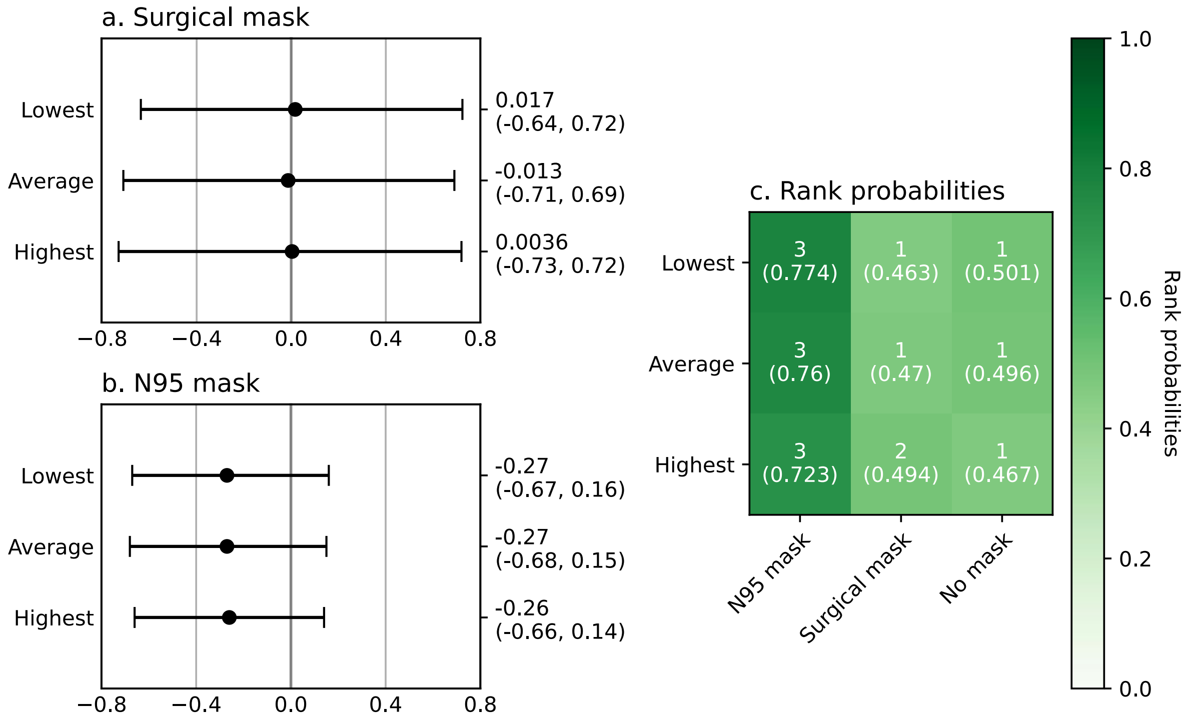

Supplementary Fig. 19. Sensitivity analysis of various imputed correlation coefficients for aural temperature. Numbers in rank probabilities heatmap represent rank with the highest probability and corresponding probability for given measure and condition

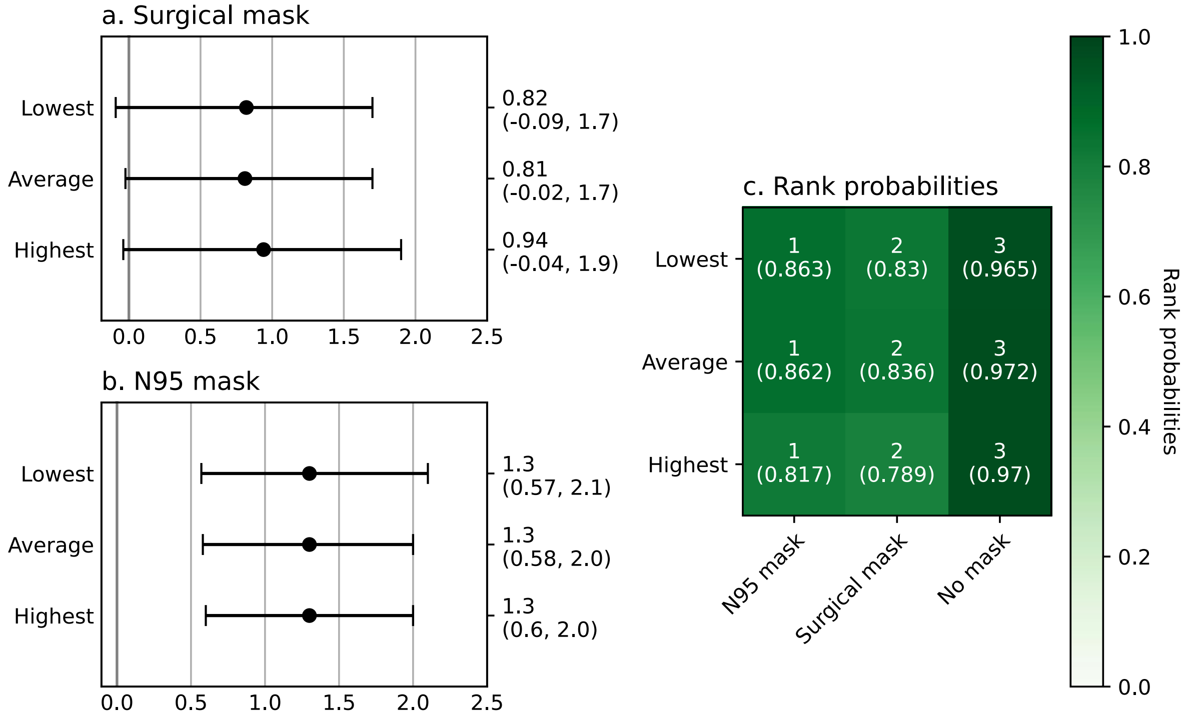

Supplementary Fig. 20. Sensitivity analysis of various imputed correlation coefficients for the temperature of facial skin covered by the mask. Numbers in rank probabilities heatmap represent rank with the highest probability and corresponding probability for given measure and condition

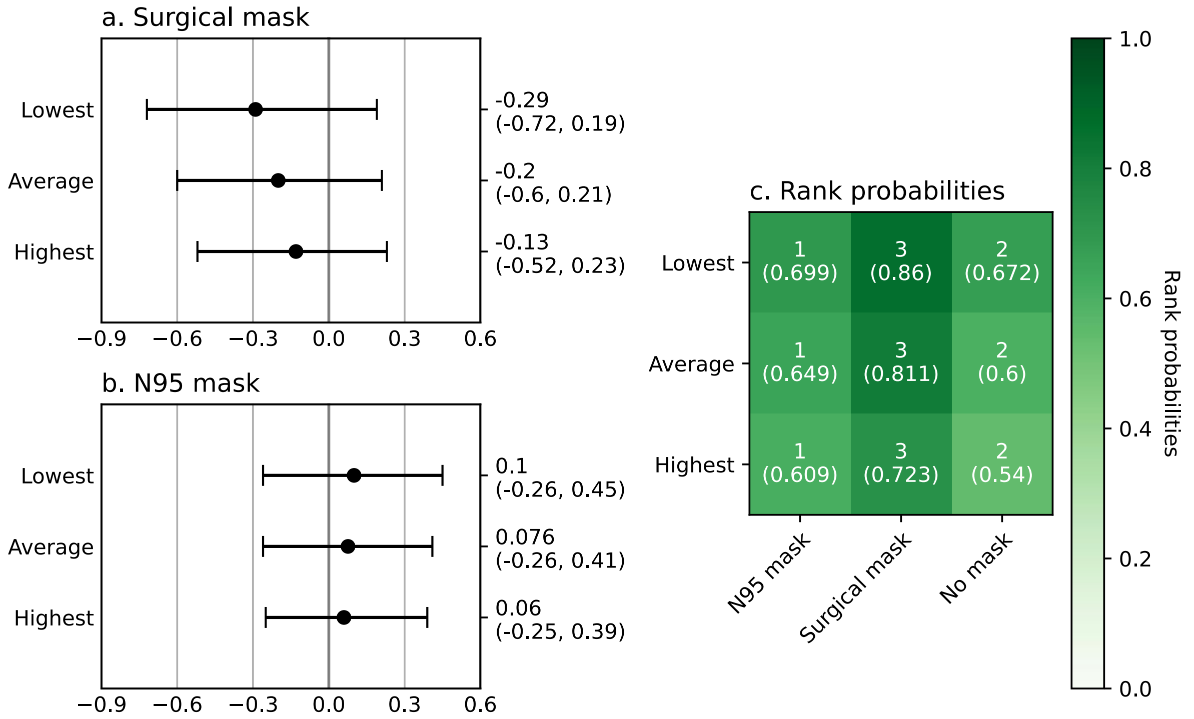

Supplementary Fig. 21. Sensitivity analysis of various imputed correlation coefficients for the temperature of uncovered facial skin temperature. Numbers in rank probabilities heatmap represent rank with the highest probability and corresponding probability for given measure and condition

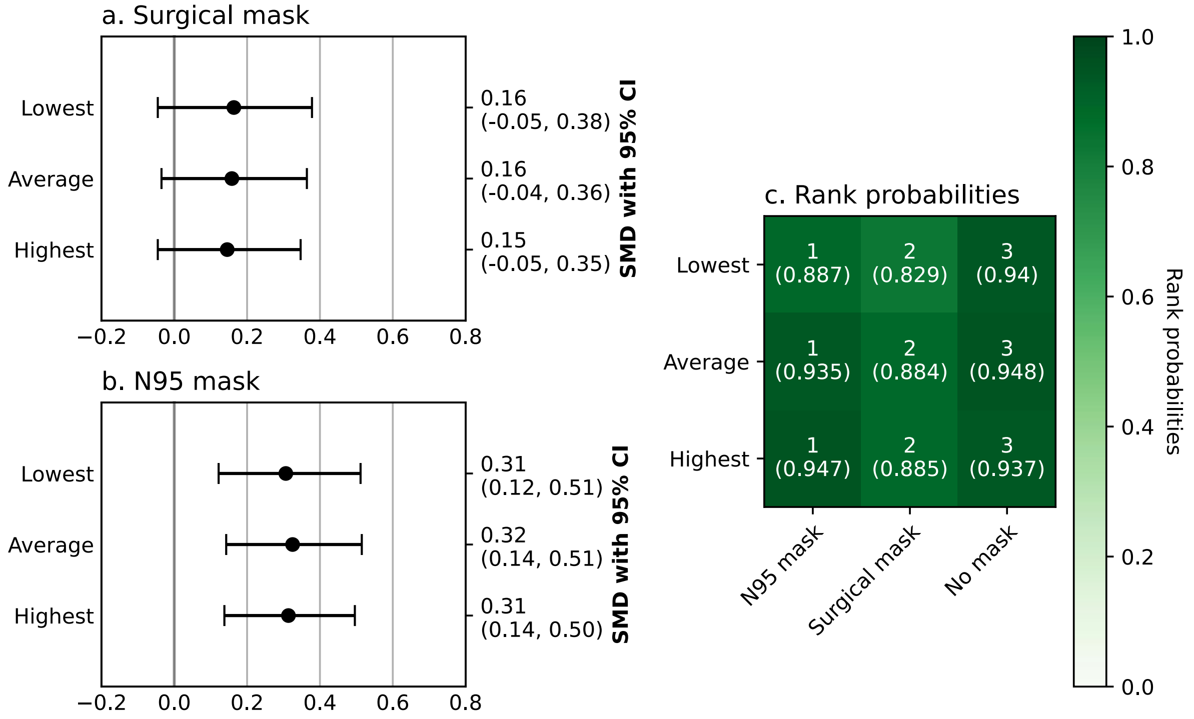

Supplementary Fig. 22. Sensitivity analysis of various imputed correlation coefficients for HR. Numbers in rank probabilities heatmap represent rank with the highest probability and corresponding probability for given measure and condition

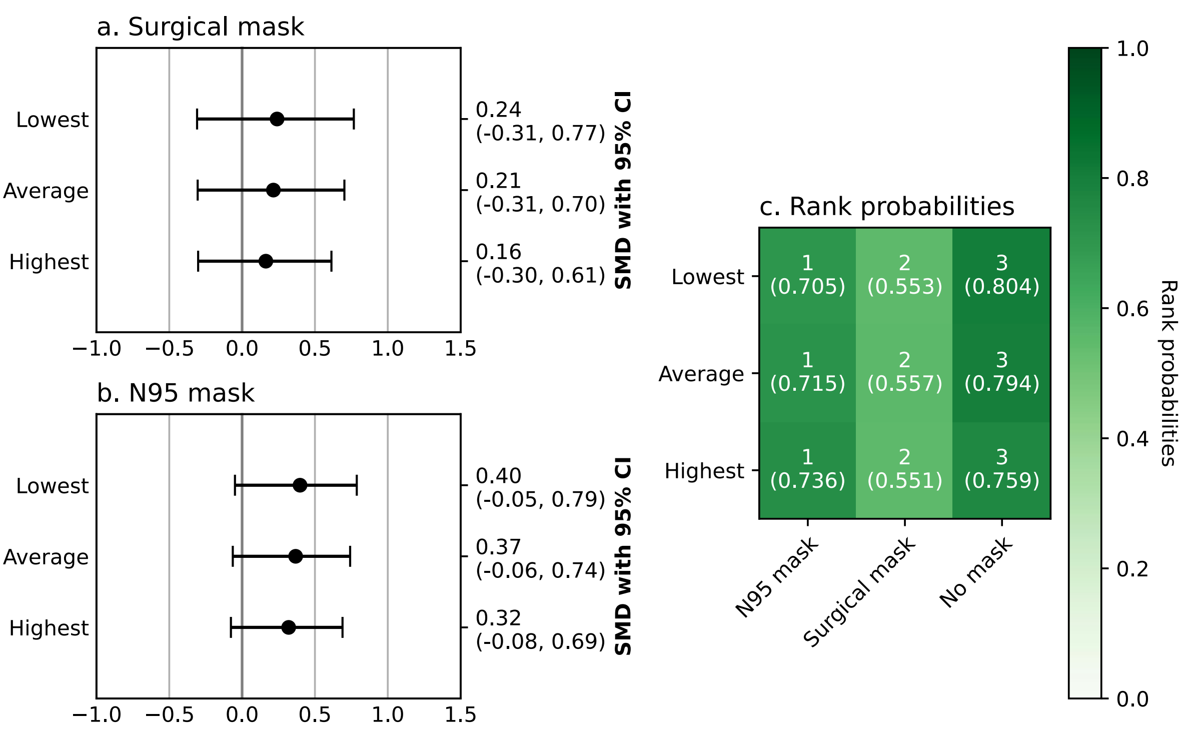

Supplementary Fig.23. Sensitivity analysis of various imputed correlation coefficients for tcPCO2. Numbers in rank probabilities heatmap represent rank with the highest probability and corresponding probability for given measure and condition

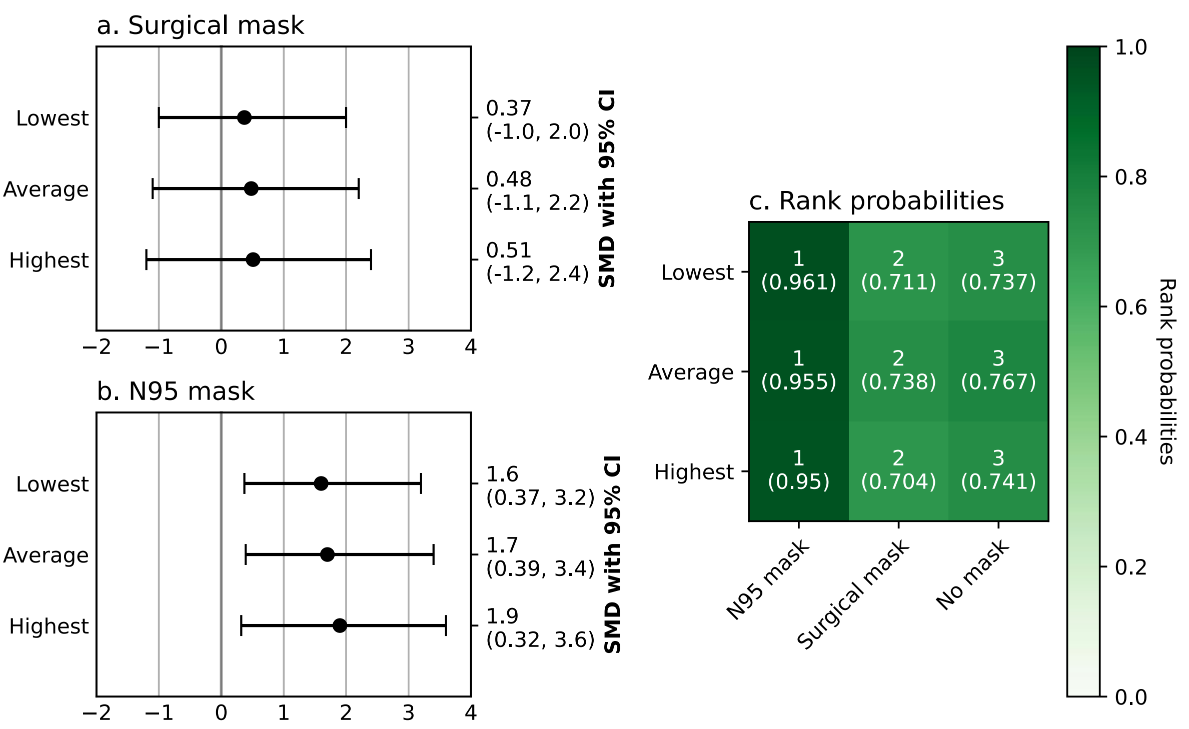

Supplementary Fig. 24. Sensitivity analysis of various imputed correlation coefficients for RHP. Numbers in rank probabilities heatmap represent rank with the highest probability and corresponding probability for given measure and condition

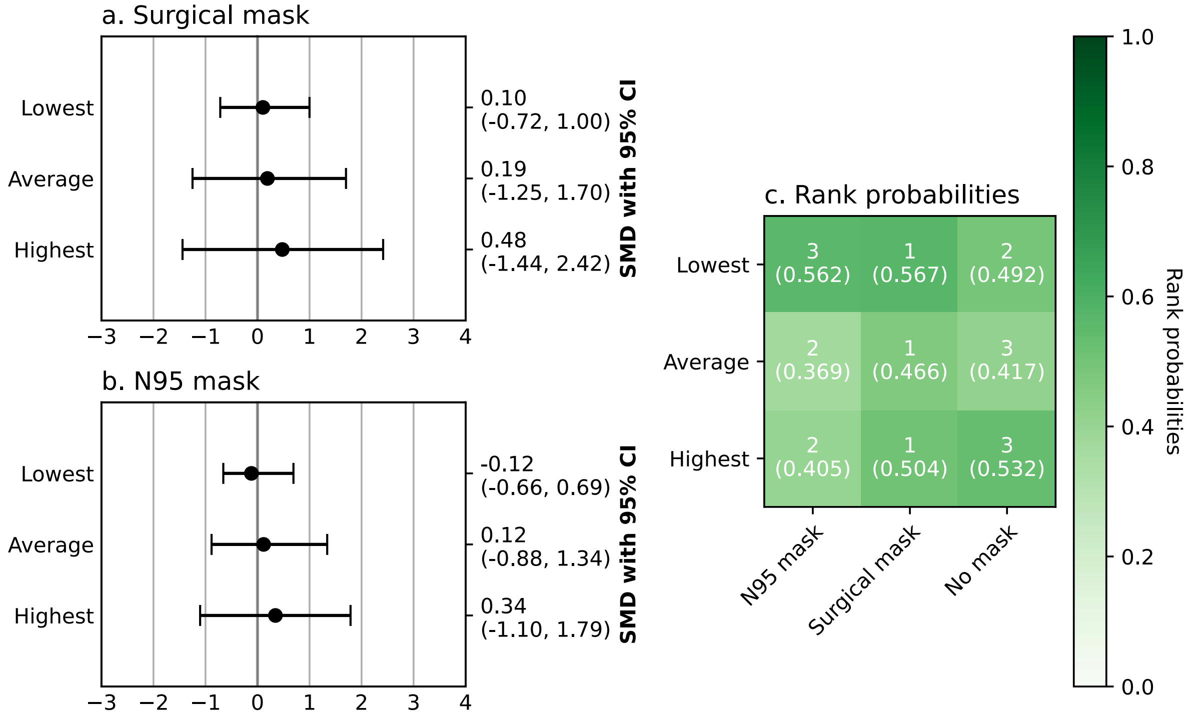

Supplementary Fig. 25. Sensitivity analysis of various imputed correlation coefficients for RR. Numbers in rank probabilities heatmap represent rank with the highest probability and corresponding probability for given measure and condition

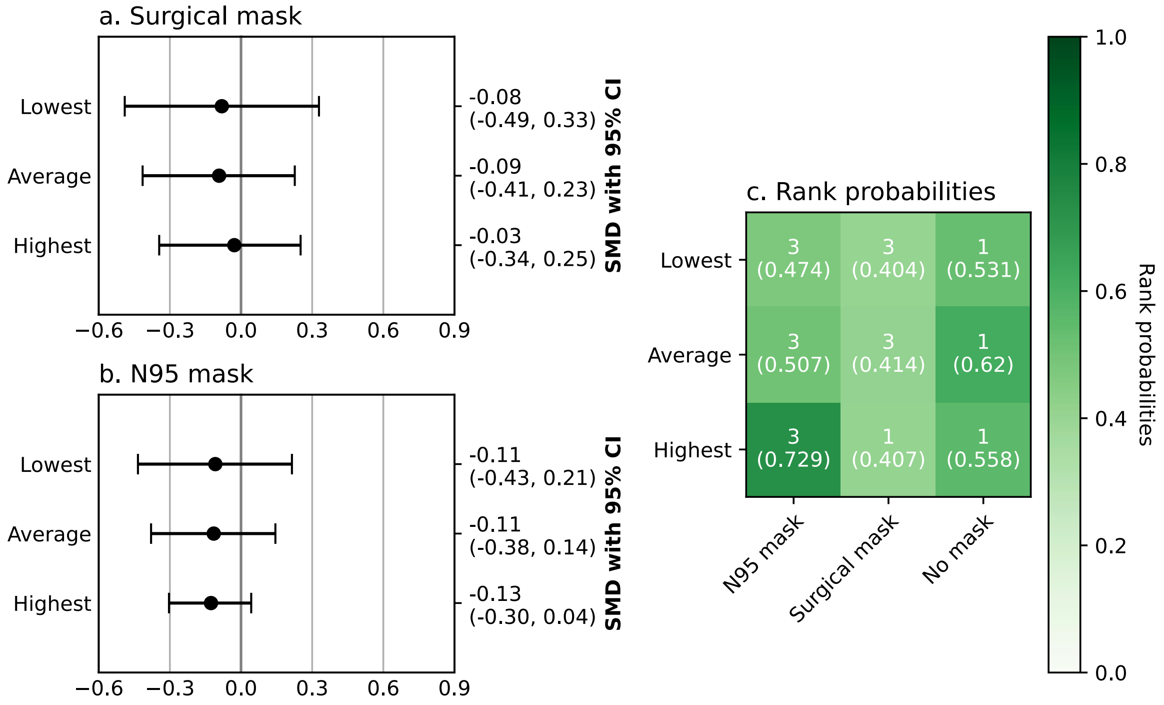

Supplementary Fig. 26. Sensitivity analysis of various imputed correlation coefficients for SpO2. Numbers in rank probabilities heatmap represent rank with the highest probability and corresponding probability for given measure and condition

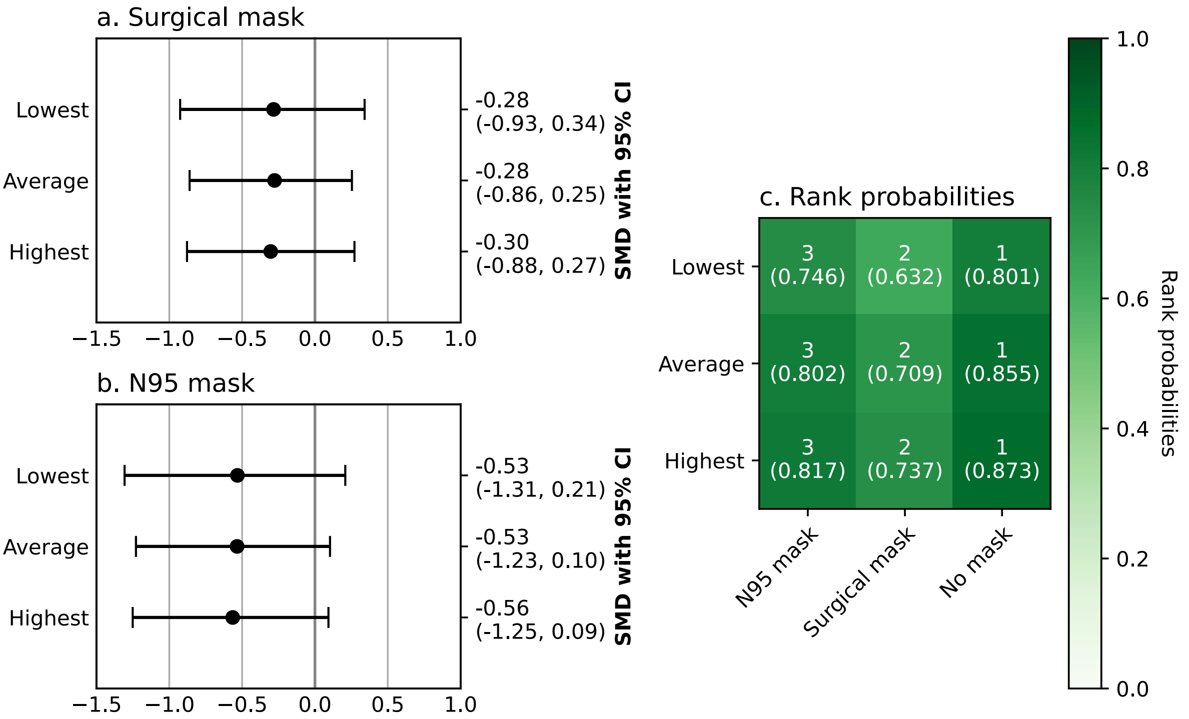

Supplementary Fig. 27. Sensitivity analysis of various imputed correlation coefficients for spO2 during high intensity. Numbers in rank probabilities heatmap represent rank with the highest probability and corresponding probability for given measure and condition

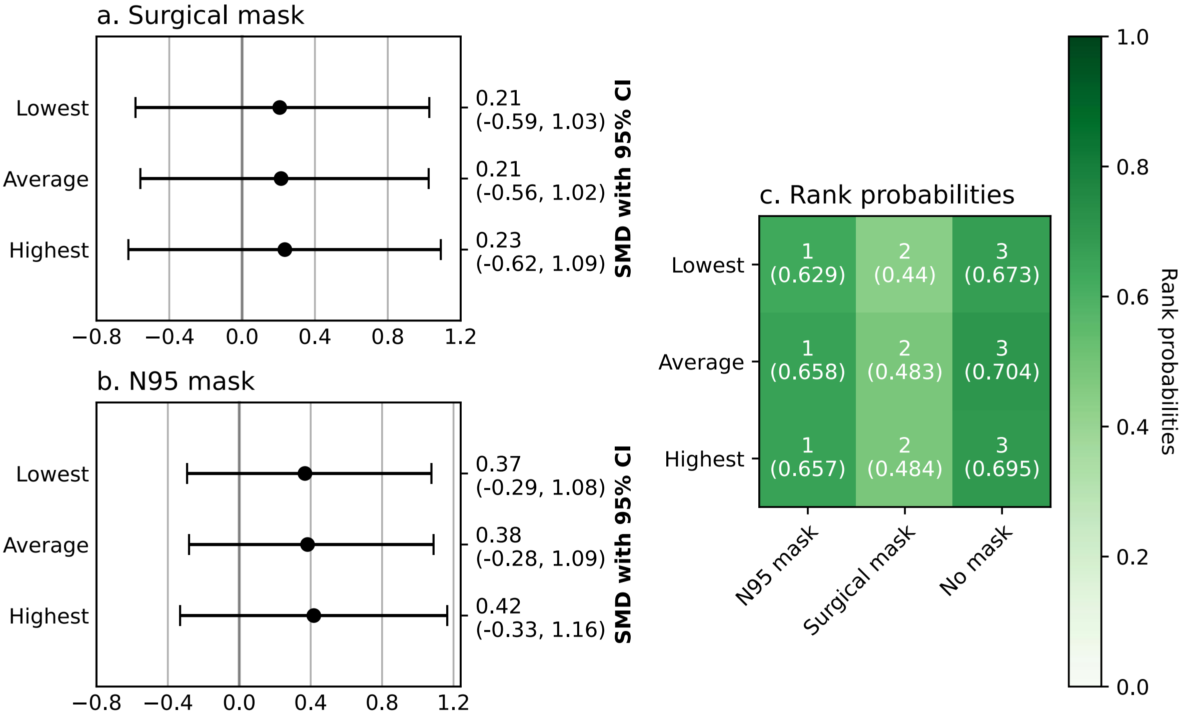

Supplementary Fig. 28. Sensitivity analysis of various imputed correlation coefficients tidal volume. Numbers in rank probabilities heatmap represent rank with the highest probability and corresponding probability for given measure and condition

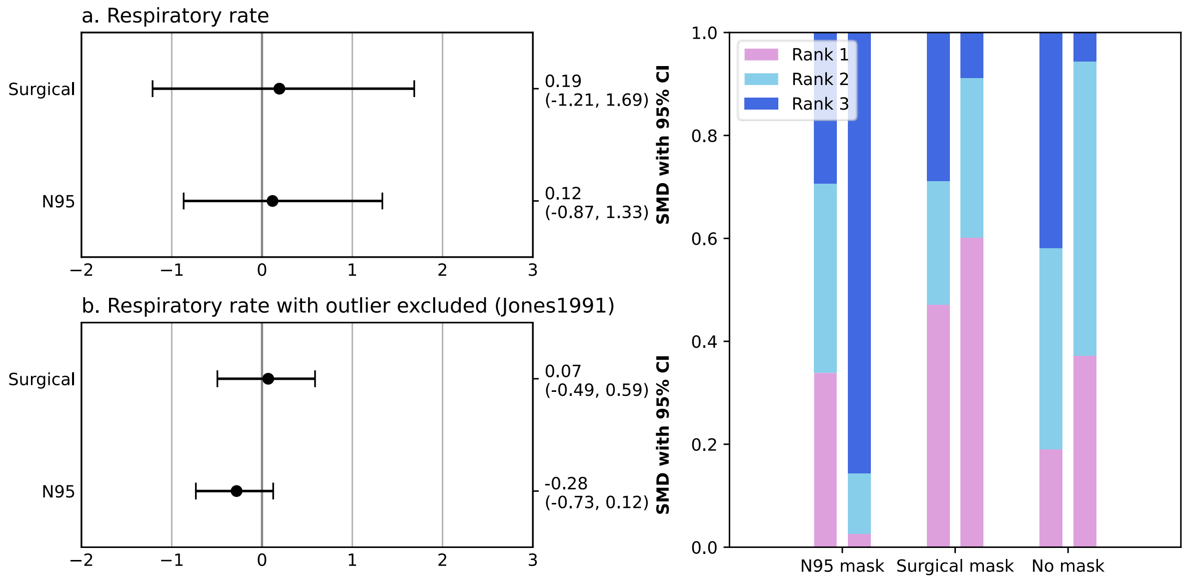

Supplementary Fig. 29. Sensitivity analysis of RR after exclusion of outlier study (rank probabilities on left – without exclusion; rank probabilities on rank – after exclusion)
